# Deblurring traffic sign images based on exemplars

**DOI:** 10.1371/journal.pone.0191367

**Published:** 2018-03-07

**Authors:** Houjie Li, Tianshuang Qiu, Shengyang Luan, Haiyu Song, Linxiu Wu

**Affiliations:** 1 Faculty of Electronic Information & Electrical Engineering, Dalian University of Technology, Dalian, Liaoning, China; 2 College of Information & Communication Engineering, Dalian Minzu University, Dalian, Liaoning, China; 3 School of Electrical Engineering and Automation, Jiangsu Normal University, Xuzhou, Jiangsu, China; 4 School of Computer Science & Engineering, Dalian Minzu University, Dalian, Liaoning, China; Mar Ephraem College of Engineering & Technology, INDIA

## Abstract

Motion blur appearing in traffic sign images may lead to poor recognition results, and therefore it is of great significance to study how to deblur the images. In this paper, a novel method for deblurring traffic sign is proposed based on exemplars and several related approaches are also made. First, an exemplar dataset construction method is proposed based on multiple-size partition strategy to lower calculation cost of exemplar matching. Second, a matching criterion based on gradient information and entropy correlation coefficient is also proposed to enhance the matching accuracy. Third, *L*_0.5_-norm is introduced as the regularization item to maintain the sparsity of blur kernel. Experiments verify the superiority of the proposed approaches and extensive evaluations against state-of-the-art methods demonstrate the effectiveness of the proposed algorithm.

## Introduction

**Fig 1:**
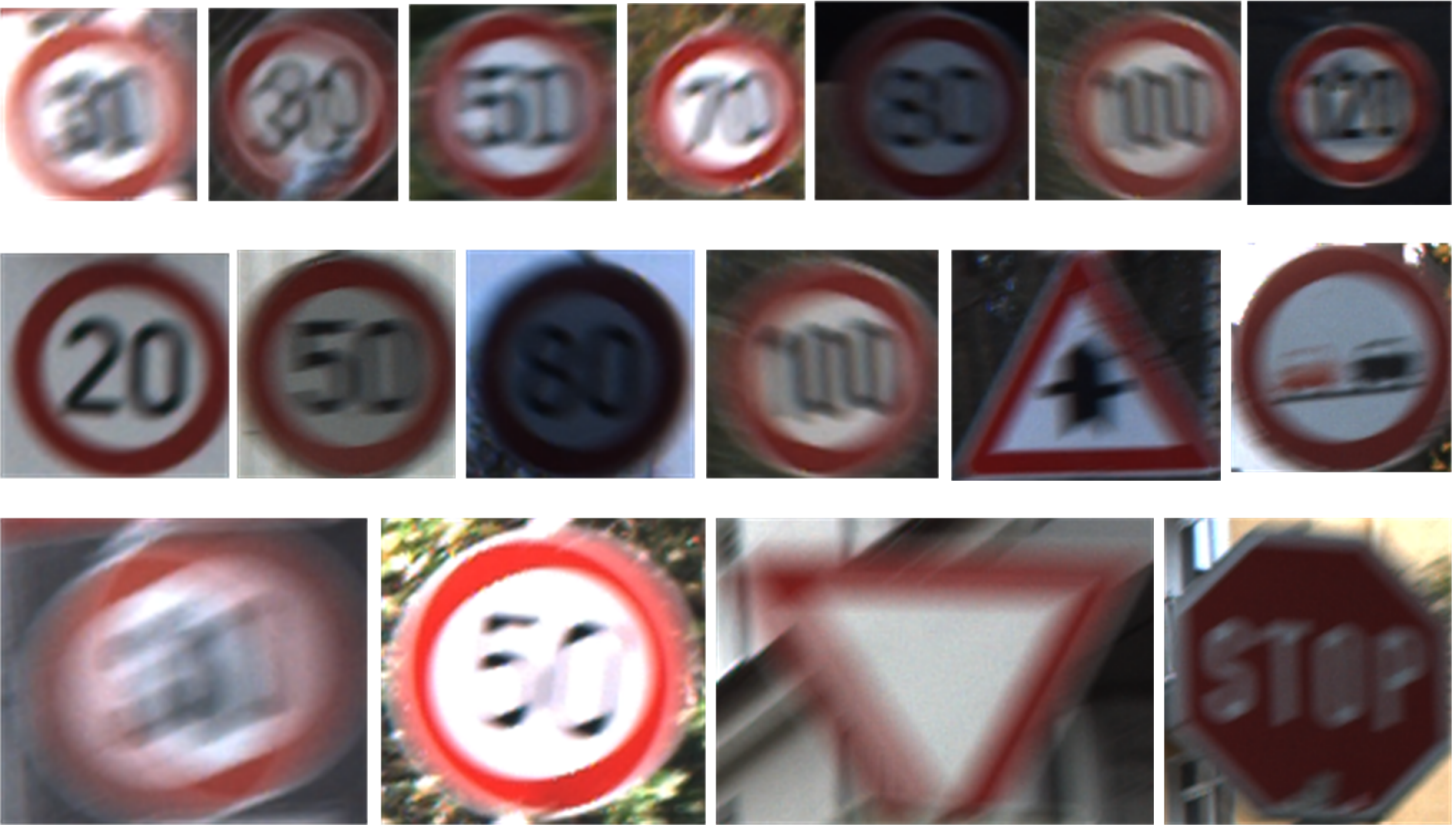
Some examples of motion blur in traffic sign images with different sizes.

Traffic sign recognition (TSR) system is an important subsystem to driver-assistant systems, automatic vehicle systems and sign inventory systems, et al. Motion blur may appear in the detected traffic sign images (Fig 1),which can cause a serious threat to later recognition and has an impact on the whole system performance [1,2,3,4]. Therefore, image recovery for the blurred traffic sign is of great importance.

Normally, deblurring traffic sign images could be considered as a blind deconvolution problem, through which, the sharp image and the blur kernel can be estimated. Under the assumption of aspatially-invariant model, the blurring procedure could be depicted as

Y=X⊗k+n (1)

where *Y* denotes the blurred image, *X* denotesthe latent sharp image, *k* denotes the blur kernel, *n* denotes the noise term and ⊗ denotes the convolution operator.

Nowadays, the methods for image deblurring could be generally categorized into four main kinds, maximum a posteriori estimation(MAP), maximum marginal distribution estimation, salient edges detection (SED) and deep learning [5,6].Among the methods mentioned above, SED method has attracted wide attentions due to superior recovering results. Within the framework of traditional MAP methods, SED method employed explicit or implicit extraction of the edge information and achieved blur kernel estimation.As a result, many SED abstraction methods are also proposed [7,8], which effectively prompted the development of image deblurring. However, SED cannot function well when salient edges do not exist since SED needs heuristic image filtering and a certain threshold value during salient edge selection procedure [9,10]. Fortunately, reference [9] proposes a deblurring method for face images based on exemplars to handle the problem of few textures mentioned above, which is exact the situation met during traffic signs deblurring since many of them share the same or at least a similar structure, but show fewer textures within the inner region and this will lead to inefficient edge information. Obviously, there still remains a void in the study of traffic sign deblurring. Meanwhile, due to the noticeable calculation cost during exemplars matching in [9], how to reduce the time cost should also be considered [11].

In this paper, a novel method of the deblurring traffic sign is proposed based on exemplars. It aims to deal with the fact that similar traffic signs have similar contours but lack rich inner textures. In order to solve this problem, first we propose an exemplar dataset construction method and it uses multiple-size partition strategy to lower the calculation time cost during exemplar matching. Second, we also propose a novel exemplar matching criterion through gradient information and the entropy correlation coefficient, and it can enhance the matching accuracy. Third, a *L*_0.5_-norm regularization item is also employed to maintain the sparsity of blur kernel. Experiment results verify the effectiveness of the proposed method.

## Related work

In recent years, many approaches have been made based on statistic prior andsalient edge, and therefore huge progress has been made in the deblurring method for a single image.

Image deblurring is considered as an ill-posed problem. In order to solve this problem, the statistic priors of natural image and blur kernel are often employed to constrain the solution. Krishnam [12] proposes a normalized sparsity prior (*L*_1_/*L*_2_-norm), which can give the true sharp solutionof the input image. Michali and Irani [13] use the similarity between image patches as the prior, which effectively characterizesthe sharp image and exhibits higher robustness. References [14–16] adopt the heavy-tailed gradient distribution prior to favor nature image since the distribution is sparse. However, these methods often introduce complicated optimization problems, which leads to high calculation complexity during kernel estimation. Reference [17] proposes a gradient prior constrained by*L*_0_-norm, which simplifies the optimization and achieves fast convergence. Reference [10] addresses a gradient prior method combined with brightness constrained by *L*_0_-normand this method can estimate blur kernel based on the reliable intermediate latent image and avoid complicated edge selection. Meanwhile, it shows superior performance in the application of text image deblurring.

After the method salient edge detection(SED)under the framework ofMAP is proposedbyJiain [18], a series of related approaches are also addressed in[7,19,20]. Joshi etal. [19] carry out blur kernel estimation through salient edgesof theblurred image. Cho et al. [20] propose anSEDmethod based on image gradient calculation, double-sideband filtering and gradient threshold value processing. Xu et al. [7] propose an automatic gradient selection method to eliminate unaccountable edge structures. These methods can be used to estimate blur kernel for images with obvious edges, but they often fail the cases of traffic signs without rich textures.

In order to solve this problem, methods based on exemplars are proposed [9, 21]. HaCohen et al. [21] propose a method which uses a sharp reference example for guidance. However, the method needs a reference image with the same content as the blurred image, which may not be practical. Panet al. [9] proposea face image deblurring method based on exemplars, which is of more practical use since it abandons the same-content requirement.However, the calculation cost for exemplar dataset is still expensive[11] and needs to be considered.

In this paper, a construction method for the exemplar dataset through multiple-size partition strategy is proposed to lower the time cost, as well as a novel matching criterion which can enhance the matching accuracy. Meanwhile, the *L*_0.5_-norm regularization item is introduced to guarantee the sparsity during blur kernel estimation.

## Proposed method

In this chapter, the traffic sign deblurring method is divided into four steps and in the first three, approaches and modifications are made to better the final recover result.

### Structure of traffic sign and dataset construction

In traffic sign dataset, the same-category traffic signs share unique contour and color. For example, in German Traffic Sign Detection Benchmark(GTSDB)[22], the prohibition signs contain the red circle contour while the warning signs have the red triangular contour. Since these contour structures areeasy to distinguish and convenient to obtain compared with inner structures, they are often chosen as the salient features for traffic signs.

According to GTSDB, the sizes of the detected signs normally vary from 16 x 16 to 128 x 128 pixels, which means the sizes of enclosing rectangular of the traffic signs are usually limited. Therefore, we can construct an exemplar dataset through multiple sized partition and then an according mask dataset as follows.

First, we need to construct the exemplar dataset.

The dataset is divided into *N* sections and each of them becomes a subset with the size

$$R_{n}=\left[v_{n}\;v_{n}+\delta_{p}\right]$$ (2)

Where *n* = 1,2,⋯,*N* denotes the subset order, *p* = 1,2,⋯,*P* denotes the multiple-size order, *P* denotes the maximum order, *v*_n_ denotes the initial size for each subset, *δ*_*p*_ denotes subset size interval. As the multiple-size order *p* increases, *δ*_*p*_ will also increase. During the exemplar matching procedure, a subset will be automatically chosen according to the size of the enclosing rectangular of the blurred sign. In this way, the matching calculation will only be carried out for the chosen subset rather than the whole dataset, which leads to significant decrease in calculation cost.

Second, we need to obtain the contour masks and construct the mask dataset.

**Fig 2:**
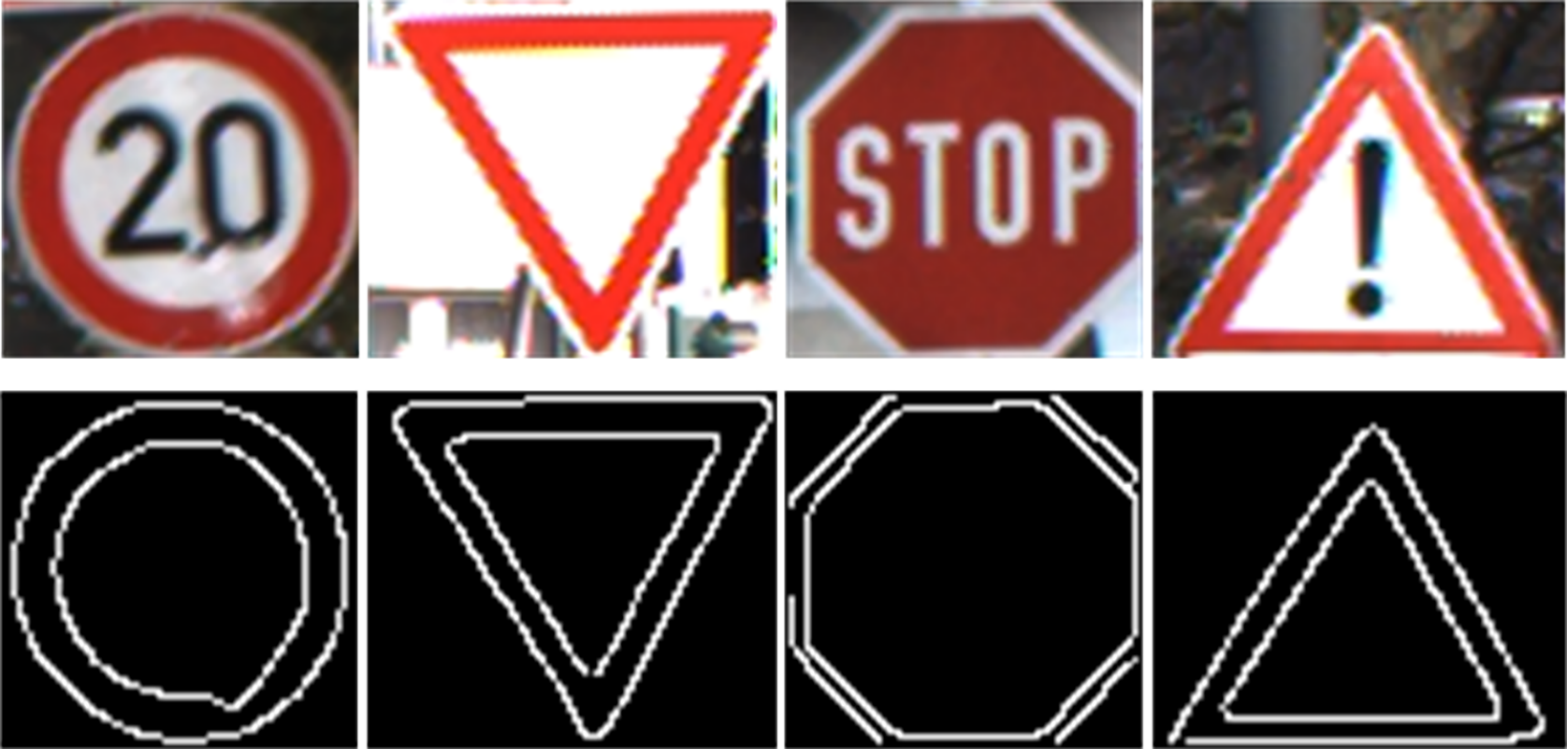
Exemplar images and the corresponding contour masks.

Initial contours are normally located manually and all the structures can be eventually obtained by using further the guided filter[23] and the Otsu method [24] to refine, referring to Fig 2.

### Exemplar matching

Generally speaking, exemplar matching means determining the most similar exemplar after comparing the blurred image with each exemplar from the dataset. Therefore, the matching criterion plays an important role in the similarity measurement.

The matching criterion proposed in the paper is named gradient information and entropy correlation coefficient matching method (GECC) since it combines the gradient information and entropy correlation coefficient (ECC). And it can be depicted as follows.

If *Y* is the test image, the ECC for exemplar can be described as [25]

$$\mathrm{ECC}\left(Y,E_{i}\right)=\frac{2\mathrm{MI}\left(Y,E_{i}\right)}{H\left(Y\right)+H\left(E_{i}\right)}$$ (3)

where *i* denotes the exemplar order, *E*_*i*_ denotes the*i*-th exemplar, H(*Y*) and H(*E*_*i*_) denote the information entropy for the blurred image and the exemplar image,respectively. MI(*Y*,*E*_*i*_) denotes the mutual information (MI) between *Y* and *E*_*i*_, and it can be described as

$$\mathrm{MI}\left(Y,E_{i}\right)=H\left(Y\right)+H\left(E_{i}\right)-H\left(Y,E_{i}\right)$$ (4)

where H(*Y*,*E*_*i*_) denotes the joint information entropy for *Y* and *E*_*i*_. Mutual information describes the statistic correlation and mutual information shared by two images. The more similar two images are, the greater the correlation and the mutual information will be.

Since image gradient contains spatial information, it can be introduced into the entropy correlation coefficient to obtain more accurate similarity matching results. The gradient information function [26] between two images could be depicted as follows

$$G(Y,E_{i})=\sum_{\left(x,x'\right)\in \left(Y\cap E_{i}\right)}\omega \left(\alpha_{x,x'}\left(\sigma \right)\right)\min\left(\left|\nabla x\left(\sigma \right)\right|,\left|\nabla x'\left(\sigma \right)\right|\right)$$ (5)

where *x*,*x*′ stand for the locations of the pixel in image *Y* and the pixel in exemplar *E*_*i*_ accordingly, ∇*x*(*σ*) and ∇*x*′(*σ*) denote the gradient vectors for *x*,*x*′ under the scalar *σ*, *α*_*x*,*x*'_(*σ*) denotes the angle between the gradient vectors of *x*,*x*′ and it can be defined by

$$\alpha_{x,x'}\left(\sigma \right)=\arccos\frac{\nabla x\left(\sigma \right),\nabla x\prime \left(\sigma \right)}{\left|\nabla x\left(\sigma \right)\right|\left|\nabla x\prime \left(\sigma \right)\right|},$$

where *ω*(*α*_*x*,*x*'_(*σ*)) is the evaluation function for two relative pixels in two images and it can be defined by 2*σ*^2^

$$\omega \left(\alpha_{x,x'}\left(\sigma \right)\right)=\frac{1}{2}\left[\cos\left(2\alpha_{x,x'}\left(\sigma \right)\right)+1\right].$$

In this way, the proposed matching measurement could be defined by

$$\mathrm{GECC}\left(Y,E_{i}\right)=G\left(Y,E_{i}\right)\mathrm{ECC}\left(Y,E_{i}\right)$$ (6)

If images *Y* and *E*_*i*_ are similar, then GECC(*Y*, *E*_*i*_) tends to be large, otherwise, it would be small. Therefore, among all the exemplars, the one which has the largest GECC(*Y*, *E*_*i*_) will be considered as the matched exemplar*T*, which means it is the most similar one to the blurred image.

∇*S* denotes the extracted salient edge from the matched exemplar and can be defined as

∇S=∇T•M (7)

where ∇*T* denotes the gradient image for *T*, *M* denotes the binary mask image forthe corresponding contour of *T* and • denotes array multiplication operator.

### Motion blur kernel estimation

In this section, we will employ the salient edge ∇*S* to estimate the motion blur kernel.Under the framework of MAP, we can recover the latent sharp image *X* and the blur kernel *k* from the blurred image *Y* according to the model below

$$\underset{X,k}{\min}{\left\|X⊗k-Y\right\|}_{2}^{2}+\lambda {\left\|\nabla X\right\|}_{0}+\gamma {\left\|k\right\|}^{\alpha }$$ (8)

where ∇*X* denotes the gradient image of the latent sharp image *X*. The first term in Eq (8) denotes the likelihood item while the second and the third are the regularization terms for ∇*X* and *k*, respectively. *λ* and *γ* are the corresponding weights.

Motion blur kernel estimation problem could be solved through alternating minimization method as shown below.

$$\underset{X}{\min}{\left\|X⊗k-Y\right\|}_{2}^{2}+\lambda {\left\|\nabla X\right\|}_{0}$$ (9)

$${\underset{k}{\min}}_{2}^{2}\left\|\nabla S⊗k-\nabla Y\right\|+\gamma {\left\|k\right\|}^{\alpha }$$ (10)

In Eq (9), *L*_0_-norm is employed to maintain the salient edge and eliminate the details from the sharp image. In Eq (10), the regularization term with *α* = 0.5 is employed to better the sparsity and stability of the blur kernel.

To solve the optimization in Eq (9), we adopt a similar method shown in [27] and employ the auxiliary variable *h* = (*h*_*x*_, *h*_*y*_) for ∇*X* and the model can be rewritten as

$$\underset{X,h}{\min}{\left\|X⊗k-Y\right\|}_{2}^{2}+\beta {\left\|h-\nabla X\right\|}_{2}^{2}+\lambda {\left\|h\right\|}_{0}$$ (11)

where *β* is a scalar weight and it increases by a factor of 2 over iterations. The solution of Eq (11) will approximate that of Eq (9) when *β* approaches infinity. In this way, we can solve Eq (11) through alternatively obtaining the solutions of *X* and *h*. And we can always obtain the intermediate latent image *X* during each iteration as follows

$$\underset{X}{\min}{\left\|X⊗k-Y\right\|}_{2}^{2}+\beta {\left\|h-\nabla X\right\|}_{2}^{2}$$ (12)

For Eq (12), we can employ fast Fourier transform (FFT) to get the optimization solution and its closed-form solution is

$$X=F^{-1}\{\frac{\bar{F\left(k\right)}F\left(Y\right)+\beta \bar{F\left(\partial_{x}\right)}F\left(h_{x}\right)+\bar{F\left(\partial_{y}\right)}F\left(h_{y}\right)}{\bar{F\left(k\right)}F\left(k\right)+\beta \bar{F\left(\partial_{x}\right)}F\left(\partial_{x}\right)+\bar{F\left(\partial_{y}\right)}F\left(\partial_{y}\right)}\}$$ (13)

where *F*(⋅) and *F*^*−*1^(⋅) denote FFT and inverse FFT, respectively, ∂_*x*_ and ∂_*y*_ denote horizontal and vertical derivative operators, accordingly and $\bar{F\left(\cdot \right)}$ denotes the complex conjugate operator.

After we get *X* according to Eq (13), we can also get the auxiliary variable *h* as follows

$$h=\left\{ \begin{array}{l} \nabla X,\;{\left|\nabla X\right|}^{2}\geq \frac{\lambda }{\beta }\\ 0,\;\mathrm{others}. \end{array}\right.,$$ (14)

We employ the Constrained Iterative Reweighed Least Square(IRLS) to solve Eq (10) [16]. Normally, the optimization problem for IRLS reduces to a quadratic programming problem, and it could be solved through the dual active-set method.

The motion blur kernel estimation can be solved as Algorithm 1.

**Algorithm 1:** Motion blur kernel estimation based on the exemplar salient edge

**Input:** Blurred image *Y*, predicted salient edges ∇*S*.

**for***ite* = 1→Ite_maxi **do**

solve for kernel *k* using Eq (10).

X←Y,*β*←2*λ*

**repeat**

solve forhusing Eq (14).

solve for*X*using Eq (13).

calculate ∇*X* for *X*.

*β*←2*β*.

**until *β*>10**^**5**^

∇*S*←∇*X*

**end for**

**Output:** Blur kernel*k* and the intermediate latent*X*.

Finally, the latent image could be estimated through a number of non-blind convolution methods when the motion blur kernel is decided. In this paper, we choose the non-blind deblurring method proposed in [28] to recover the latent image.

## Experiment and analysis

To verify the superiority of the proposed method, we will carry out first three experiments focusing on evaluating the matching time cost, matching accuracy and blur kernel sparsity and then two experiments mainly focusing on the overall results for synthetic images and real images.

Evaluative experiments are performed in MATLAB on an Intel Core i3-4150 CPU with 4 GB RAM. In all experiments, the parameters *λ*, *γ* and Ite_maxi are set to be 0.002, 0.01 and 50, respectively.

### Experiment 1: Matching time cost comparison

(1) Parameters setting and the related guidance for exemplar dataset construction

In the proposed construction method for the exemplar dataset, the entire exemplar dataset is divided into multiple subsets. Instead of equal-size intervals, multiple-size intervals are employed here according to the fact that the size of interval has more compact on smaller size subsets than larger ones.

Constructing the exemplar dataset from German Traffic Sign Recognition Benchmark (GTSRB) [29] with *N* = 13 and *P* = 4, we can get 3 subsets, when the sizes are within the range [1630] with *δ*_1_ = 5, 3 subsets when [3158] with *δ*_2_ = 7, 4 subsets when [5988] with *δ*_3_ = 10, 3 subsets when [89128] with *δ*_4_ = 13.

In this way, the dataset is divided into 13 subsets, which will be selected respectively according to the size of the enclosing rectangular of the blurred traffic sign during exemplar matching. For each subset, 50 exemplars will be selected and a total of 650 exemplars will be obtained for the whole dataset.

(2) Experiment subjects and experiment progress

10 clear images are collected within the size range [7075], including 2 the speed limit signs of 20 kilometers per hour (KPH), 4 signs with 30 KPH, 2 give-way signs and 2 stop signs.Each image is blurred with 8 different kernels and 80 test images are obtained.

Maximum response of normalized cross-correlation (NCC) will be employed as the measurement tool for both methods.

Experimental results about the matching calculation time cost are shown in Table 1.

**Table 1:** Matching calculation cost comparison.

methods	Testimages	Exemplars involved in matching	Tatol calculation time(s)	Everage time of each image(s)
**[9]**	80	650	9380.7667	117.2599
**This paper**	80	50	662.0180	8.2752

Accordingto Table 1, the performance of the proposed method surpasses [9] in the calculation speed. The main reason is that the size of the subset involved in matching decreases significantly compared with the original dataset used by [9].

### Experiment 2: Matching accuracy comparison

To verify the accuracy of the proposed method, some classic methods, such as normalized cross-correlation (NCC),mutual information (MI) and entropy correlation coefficient (ECC), are selected as the comparing opponents.

50 pairs of images are selected from GTSRB. The images in each pair have the same traffic sign but slightly different size and background. Half of the images are used as the exemplar dataset, and the other half is used as the test set. Each image from the test set will be processed by 8 blur kernels provided in reference [14] and 11 noiselevels (0% - 10%).

In this experiment, success ratio is utilized as the measurement and the result is considered to be matching only when the matched exemplar is the corresponding one in the test set. Success ratio r_s_ is defined as follows

$$r_{s}=\frac{N_{m}}{N_{t}}\times 100\%$$ (15)

where *N*_t_ is the total number of the test images and *N*_m_ is the number of the successfully matched images.

The results of success ratio are shown in Table 2.

**Table 2:** Matching accuracy comparison.

Matching methods	*N*_t_	*N*_m_	r_s_
**NCC**	4400	4303	97.80%
**MI**	4400	3526	80.13%
**ECC**	4400	3789	86.11%
**GECC**	4400	4354	98.95%

According to Table 2, the proposed GECC outperforms all the other three methods.

**Fig 3:**
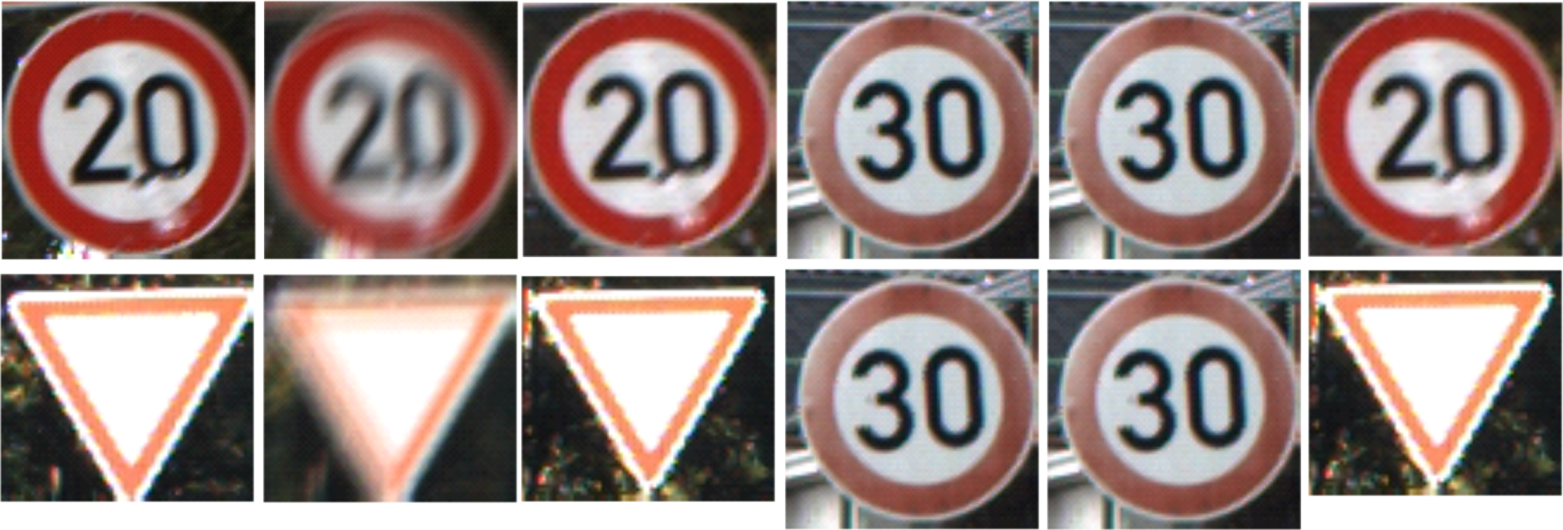
Matching results comparison. (a)—(f) denote sharp image, the corresponding blurred image, NCC result, MI, ECC and our method, respectively, about an example of speed limit 20 signs. (g)—(l) denote sharp image, the corresponding blurred image, NCC, MI,ECC and our method, respectively, about another example of give way signs.

Besides the statistic results, visual results are also shown in Fig 3.

It is obvious that in both examples shown in Fig 3, GECC and NCC succeed in the matching results while MI and ECC fail, which coincide with the statistic results shown in Table 2 that both GECC and NCC have higher accuracy than MI and ECC.

MI is very sensitive to the overlap between images and ECC is derived from MI. Both MI and ECC neglect the spatial information contained in the images, which means that a randomreshuffling of the image voxels(identical for both images) yields the same ECC or MI value as for the originalimages.And this may lead to the dilemma for exemplar matching that a shuffled image may have larger MI or ECC value than the most matching one.On the other hand, GECC employs the gradient information and successfully avoids the dilemma mentioned above.

### Experiment 3: Kernel sparsity comparison

To verify the sparsity of the blur kernels estimated, experiments are conducted between the proposed method and the method in [9]. Since kernels have sparse features, sparsity is measured in terms of kernel similarity [9].

**Fig 4:**
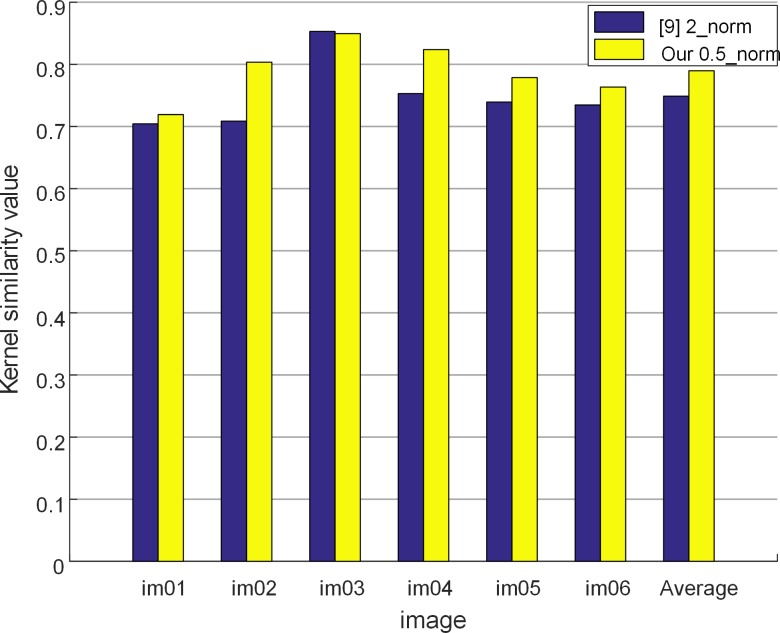
Kernel similarity comparison.

6 images are selected from dataset GTSRB and they are blurred by 8 different blur kernels, resulting in 48 test images. Results are shown in Fig 4.

According to Fig 4, our method using*L*_0.5_-norm exhibits better similarity compared with [9] using *L*_2_-norm in general.

**Fig 5:**
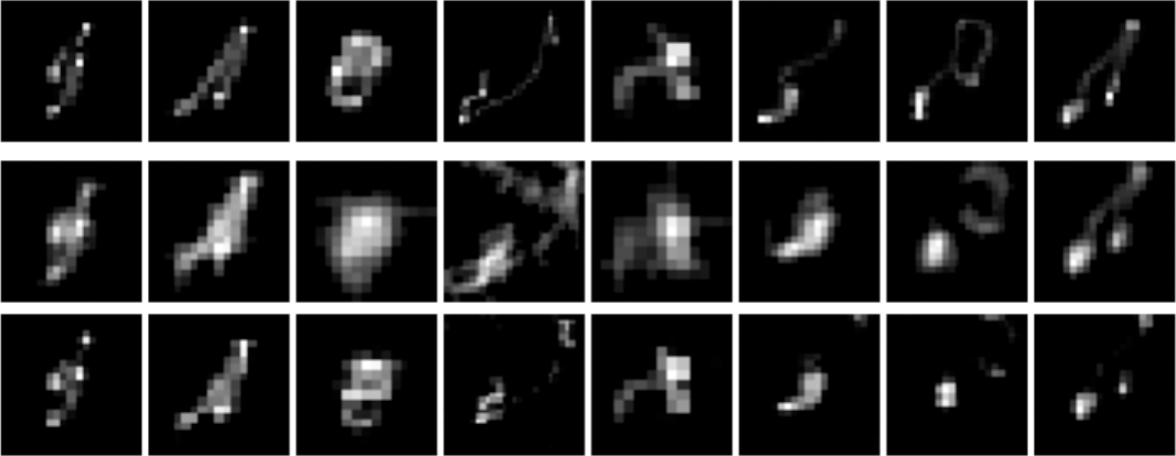
Sparsity comparison. (a)Real blur kernel (b) estimated kernel with L2-norm (c) estimated kernel with L_0.5_-norm (the true kernel is provided in [14]).

Visualresults for different norms can be found in Fig 5.

From Fig 5, we can find that kernels estimated by our method tend to be more sparse than those by [9], and meanwhilecompared with the real blur kernel, our kernels are more similar.

In the blur kernel estimation model from reference [9], *L*_2_ norm is utilized, which can guarantee the stability but not describe the sparsity of blur kernel. According to [16], we can achieve better sparsity when the norm is smaller than 1, which gives us the reason to employ a better norm,for example, 0.5, in our method. However, better sparsity also comes at the price at higher calculation complexity.

### Experiment 4: Recovery evaluation forsyntheticdataset

To verify the recovery performance of the proposed method, we evaluate it against state-of-the-art methods, such as SED[7], sparsity prior [12, 15,16,17] and exemplars[9]. The codes used are all provided by original paper authors just for fair. During image recovering,weadoptthe non-blind deconvolution method from [28] with same parameters.

50 images are selected from GTSRB and blurred by 8 different blur kernels, which generates 400 test images.

Error ratio *r*_error_ is initially presented in reference [14] and it is explained as follows

$$r_{\mathrm{error}}=\mathrm{std}\left(I_{\mathrm{ek}},I_{t}\right)/\mathrm{std}\left(I_{\mathrm{tk}},I_{t}\right)$$ (16)

Where *I*_ek_ and *I*_tk_ denote recovered images through the estimated kernel and true kernel respectively. *I*_t_ denotes true image. std(⋅) denotes the standard deviation.

Under a certain error ratio, the success rate denotes the ratio between the number of tested images below this error ratio and the total number of tested images.

**Fig 6:**
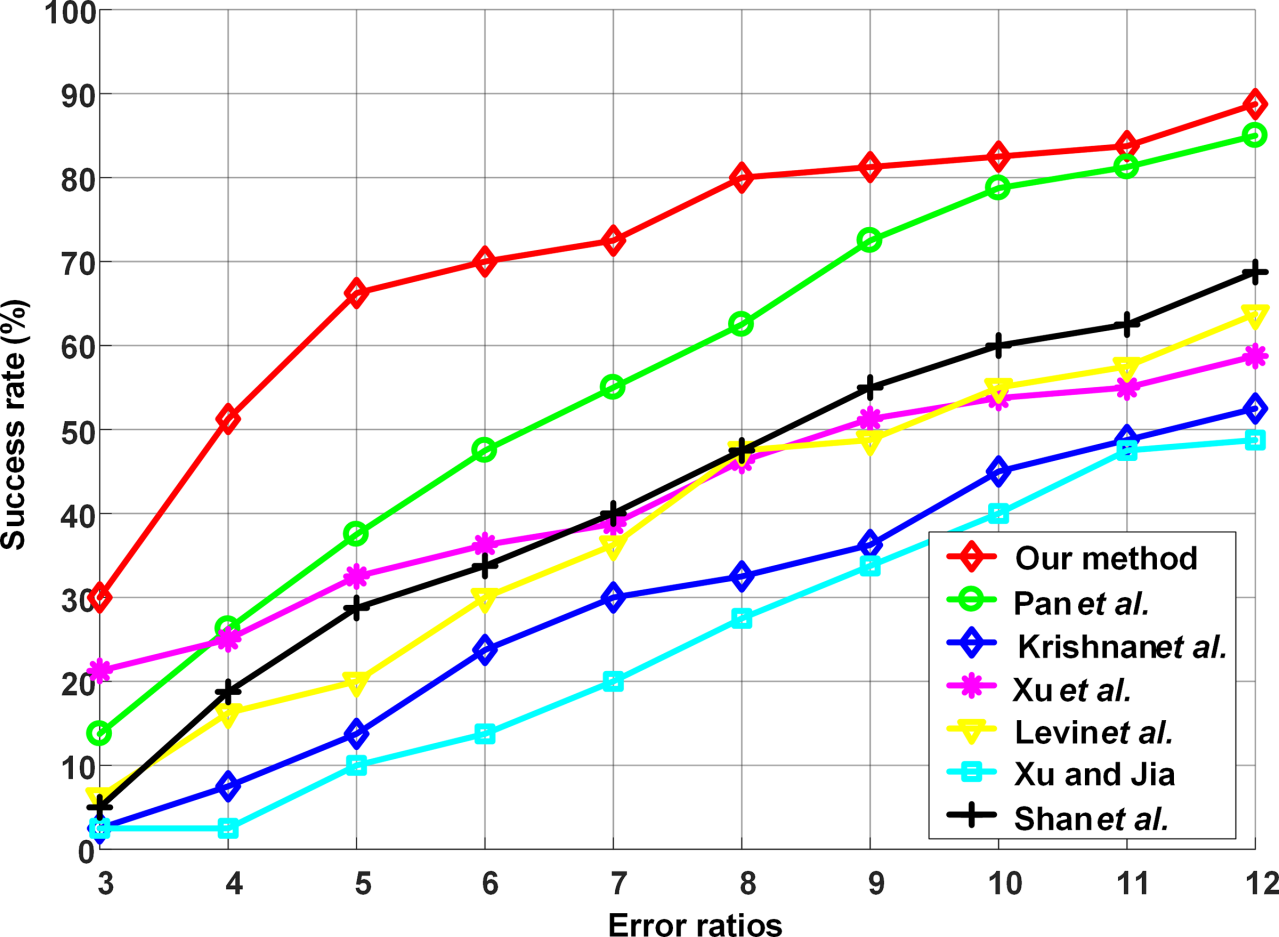
Quantitative comparisons among blind deblurring methods.

Obviously, under a certain error ratio, the lager success rate is, the better recovered image quality will be.The cumulativeerror ratios are shown in Fig 6.

From Fig 6 we can see that the curve of the proposed method is higher than other curves in general, demonstrating its better performance. Therefore, our methodoutperforms other state-of-the-art methods in synthetic traffic image deblurring.

**Fig 7:**
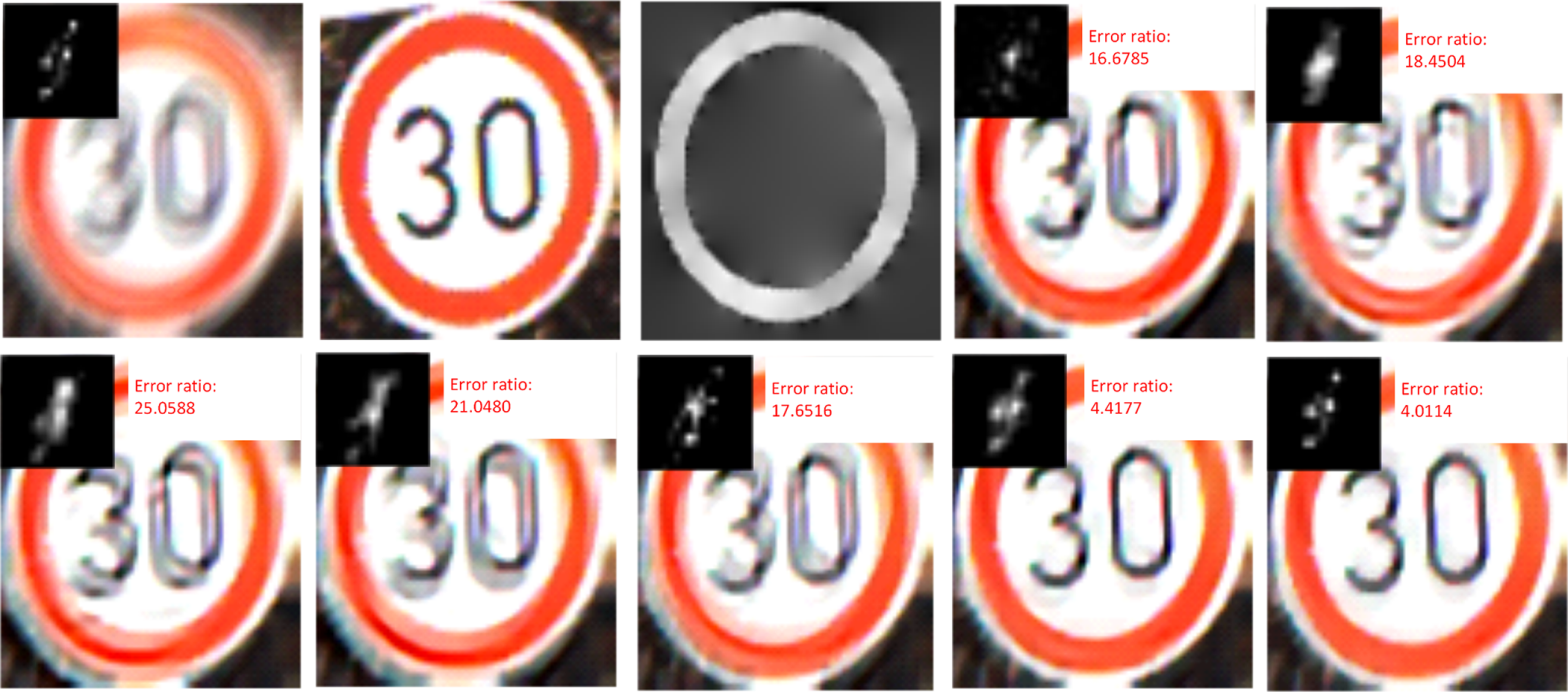
Image deblurring comparison for synthetic images. (a) denotes the input image and real blur kernel. (b) denotes the matched exemplar. (c) denotes the predicted ∇*S*. (d)—(j) denote the deblurring results of Krishnan[12], Xu eta.[17], Shan[15], Xu & Jia[7], Levin[16], Pan[9] and our method, respectively. In (d)—(j), each image includes deblurring image, estimated kernel and error ratio.

A visual exampleand according error ratios can be found in Fig 7.

We can find through the proposed method, we can get a clearer contour structure in the recovered image and a more similar blur kernel. We can also find that the SED based deblurring method [7] and the sparsity prior based methods [12,16,15,17]result in obvious artificial effect, since those based on sparsity prior fail to describe the contour features of the traffic signs and those based on SED cannot obtain reliable salient edge information from the blurred traffic sign images.

**Fig 8:**
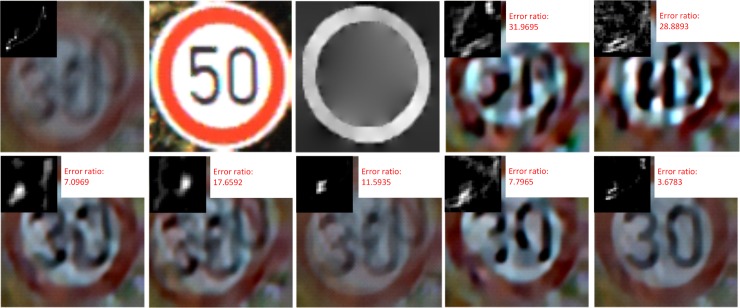
Deblurring example suffering from a heavier blur. (a) denotes the input image and real blur kernel. (b) denotes the matched exemplar. (c) denotes the predicted ∇*S*. (d)—(j) denote the deblurring results of Krishnan[12], Xu eta.[17], Shan[15], Xu & Jia[7], Levin[16], Pan[9] and our method, respectively. In (d)—(j), each image includes deblurring image, estimated kernel and error ratio.

To further verify the effectiveness of the proposed method, another example, suffering an even worse blur, is given in Fig 8.

Fig 7(B) and Fig 8(B) demonstrate the best matched exemplars of two examples. The inner structure of the matched exemplar for Fig 7 is slightly different from that of the test image, while the exemplar of Fig 8 and the test images have different traffic signs, but similar contour structures. From Fig 8 we can see that blur kernel still can be effectively estimated through the proposed method even though there are only similar contours but different inner structures.

### Experiment 5: Recovery evaluation for real images

**Fig 9:**
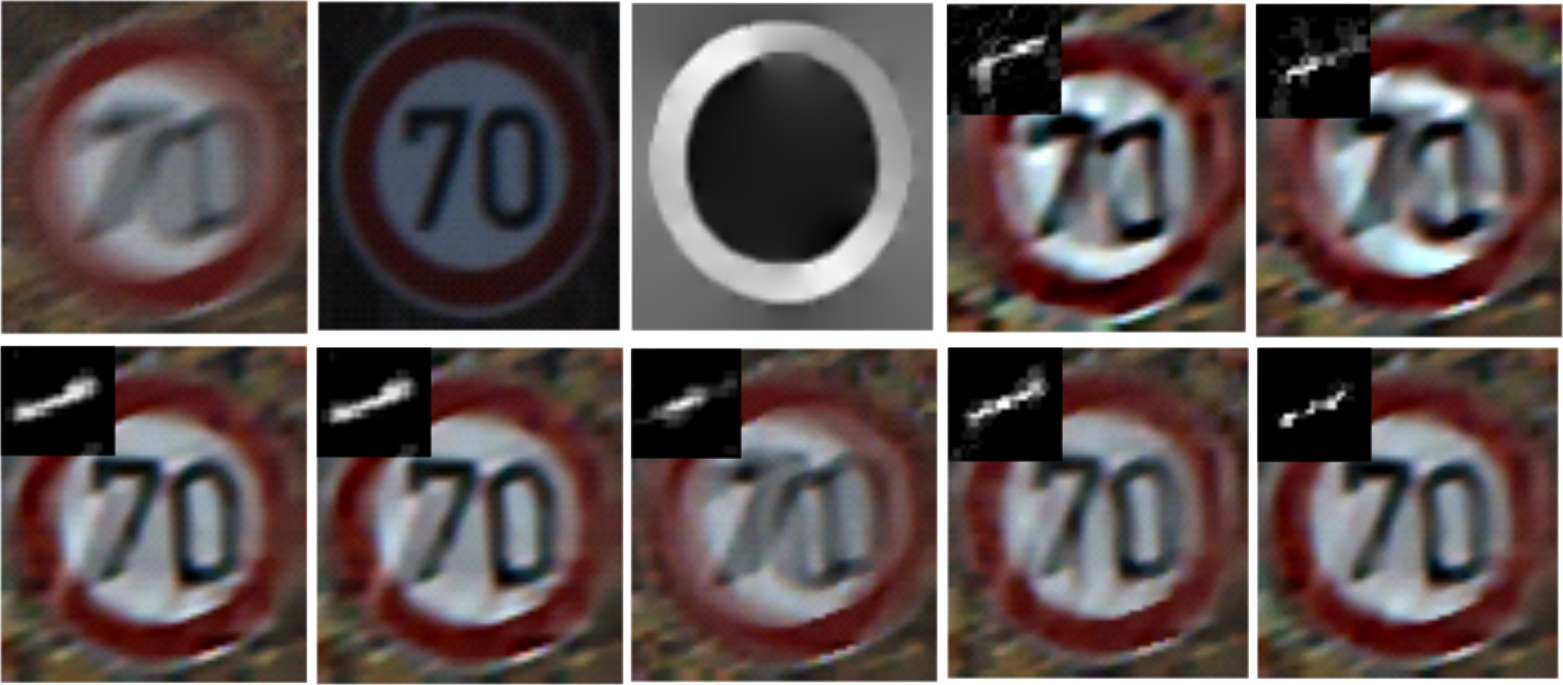
Example of real captured image. (a) denotes the real captured image. (b) denotes the matched exemplar. (c) denotes the predicted ∇*S*. (d)—(j) denote the deblurring results of Krishnan[12], Xu eta.[17], Shan[15], Xu & Jia[7], Levin[16], Pan[9] and our method, respectively. In (d)—(j), each image includes deblurring image and estimated kernel.

In this part, real traffic sign images are employed to verify the effectiveness of the proposed method.Fig 9 illustrates the deblurring results for a real traffic sign image.

From Fig 9, it is obviously shown that methods from references [7, 9, 15] still have artificial effect while those from [12,16,17] result in observant distortion and blurring. Our proposed method outperforms these methods in deblurring results.

### Experiment 6: Recovery evaluation for synthetic images with different scenarios

**Fig 10:**
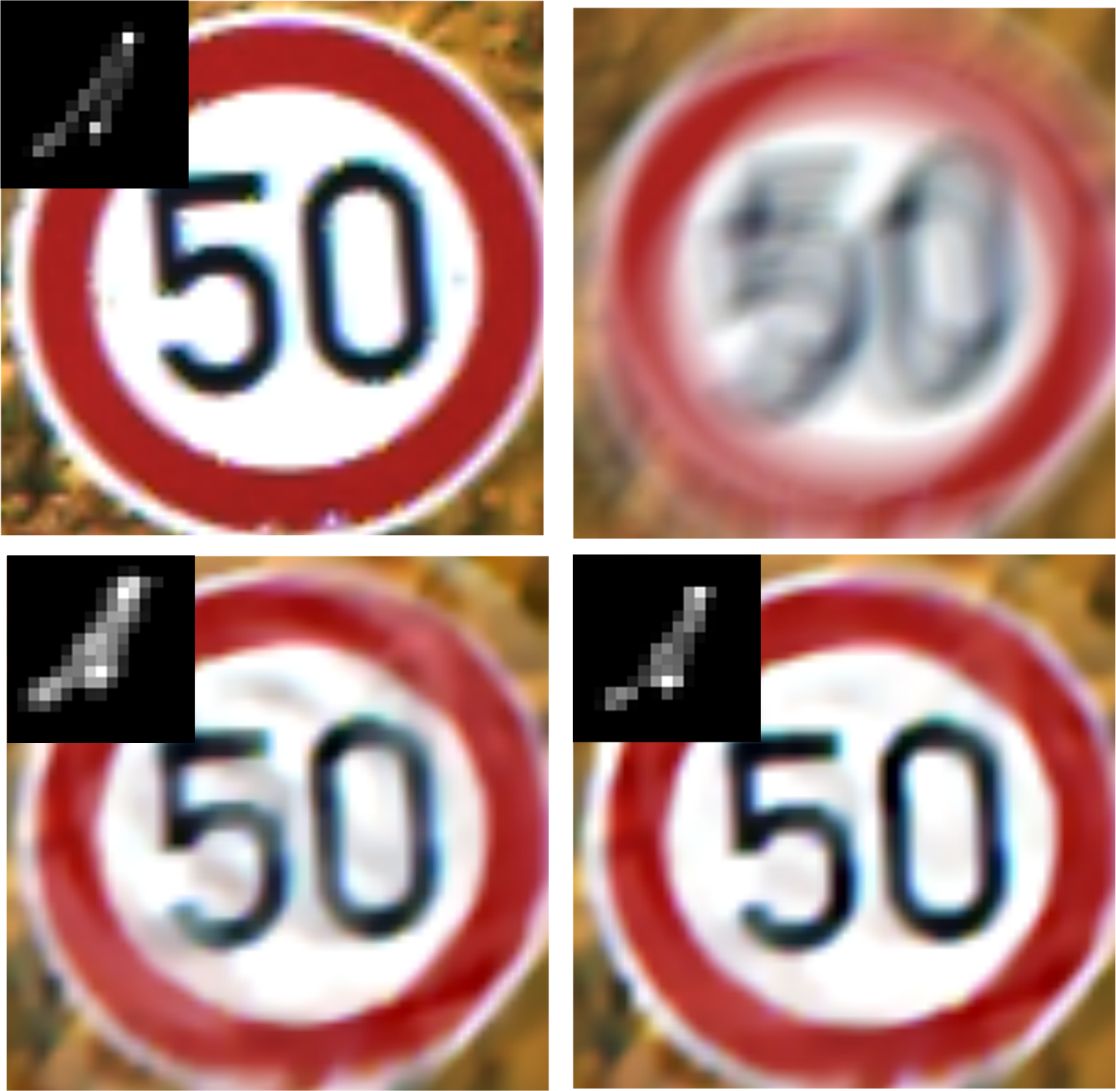
Deblurring results for synthetic traffic signs during sunny day time. (a) denotes the input image and real blur kernel. (b) denotes the blurred image. (c) and (d) denote the deblurring results of Pan[9] and our method, respectively.

**Fig 11:**
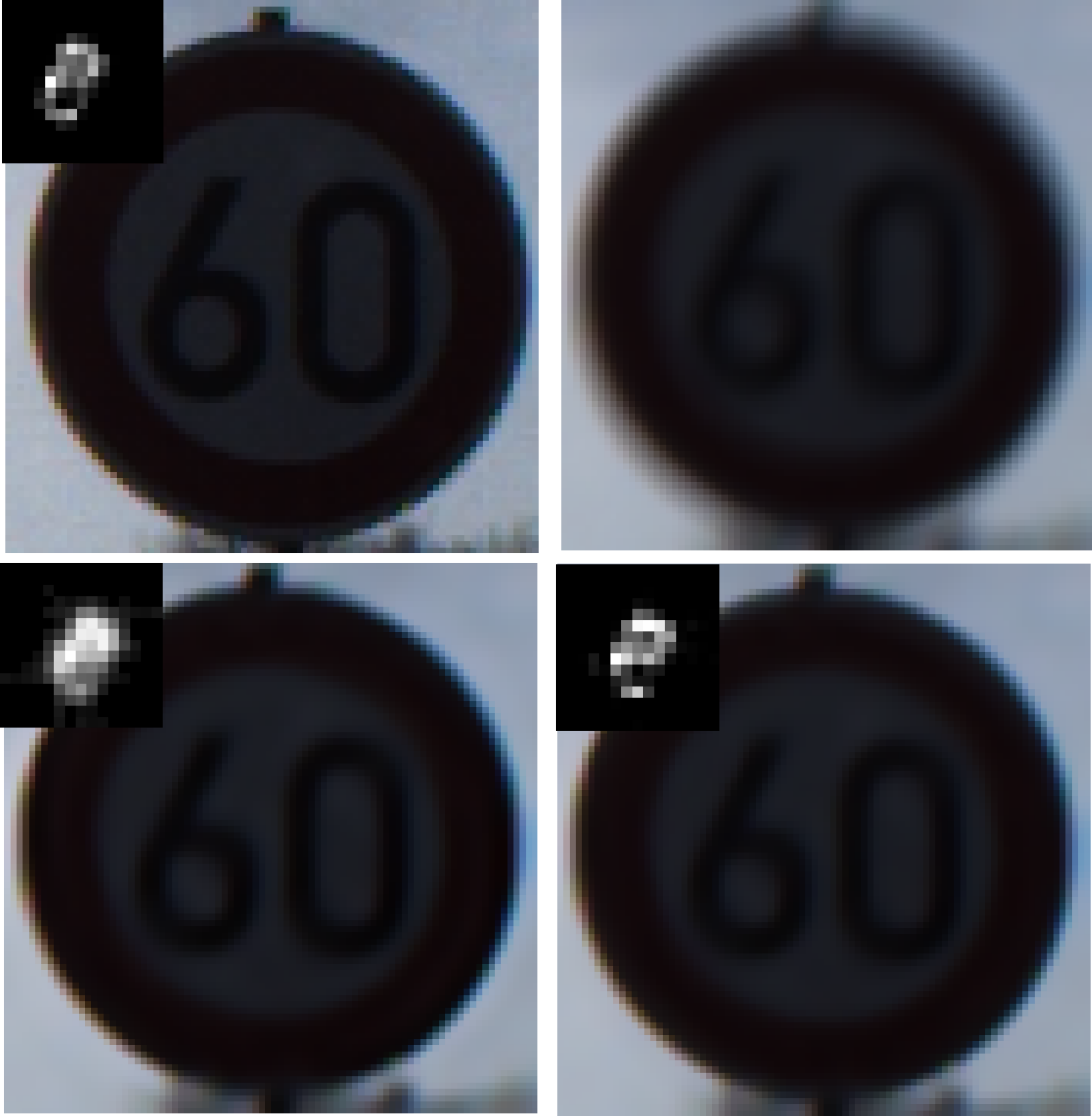
Deblurring results for synthetic traffic signs during cloudy day time. (a) denotes the input image and real blur kernel l. (b) denotes the blurred image. (c) and (d) denote the deblurring results of Pan[9] and our method, respectively.

**Fig 12:**
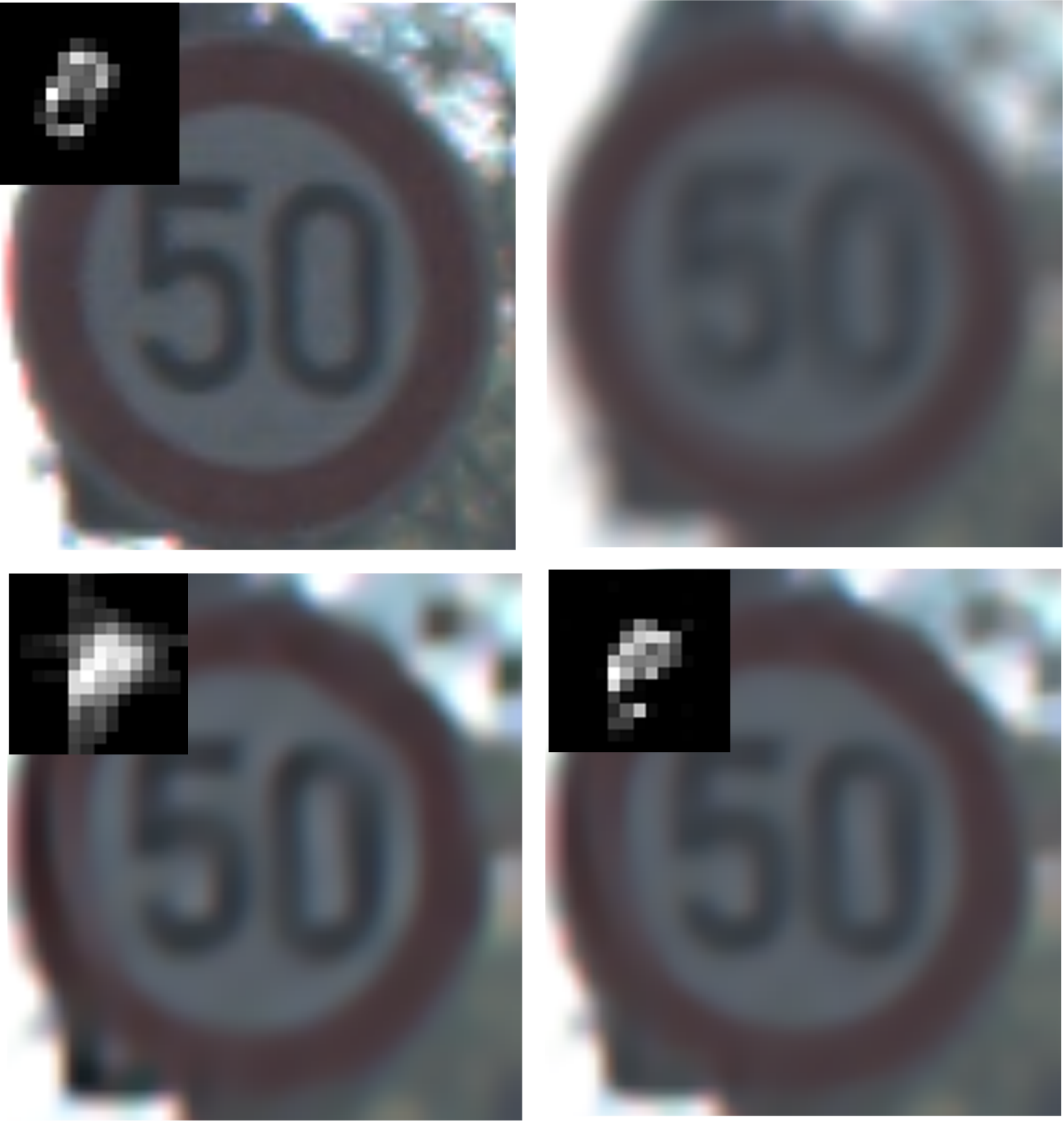
Deblurring results for synthetic traffic signs during foggy day time. (a) denotes the input image and real blur kernel. (b) denotes the blurred image. (c) and (d) denote the deblurring results of Pan[9] and our method, respectively.

**Fig 13:**
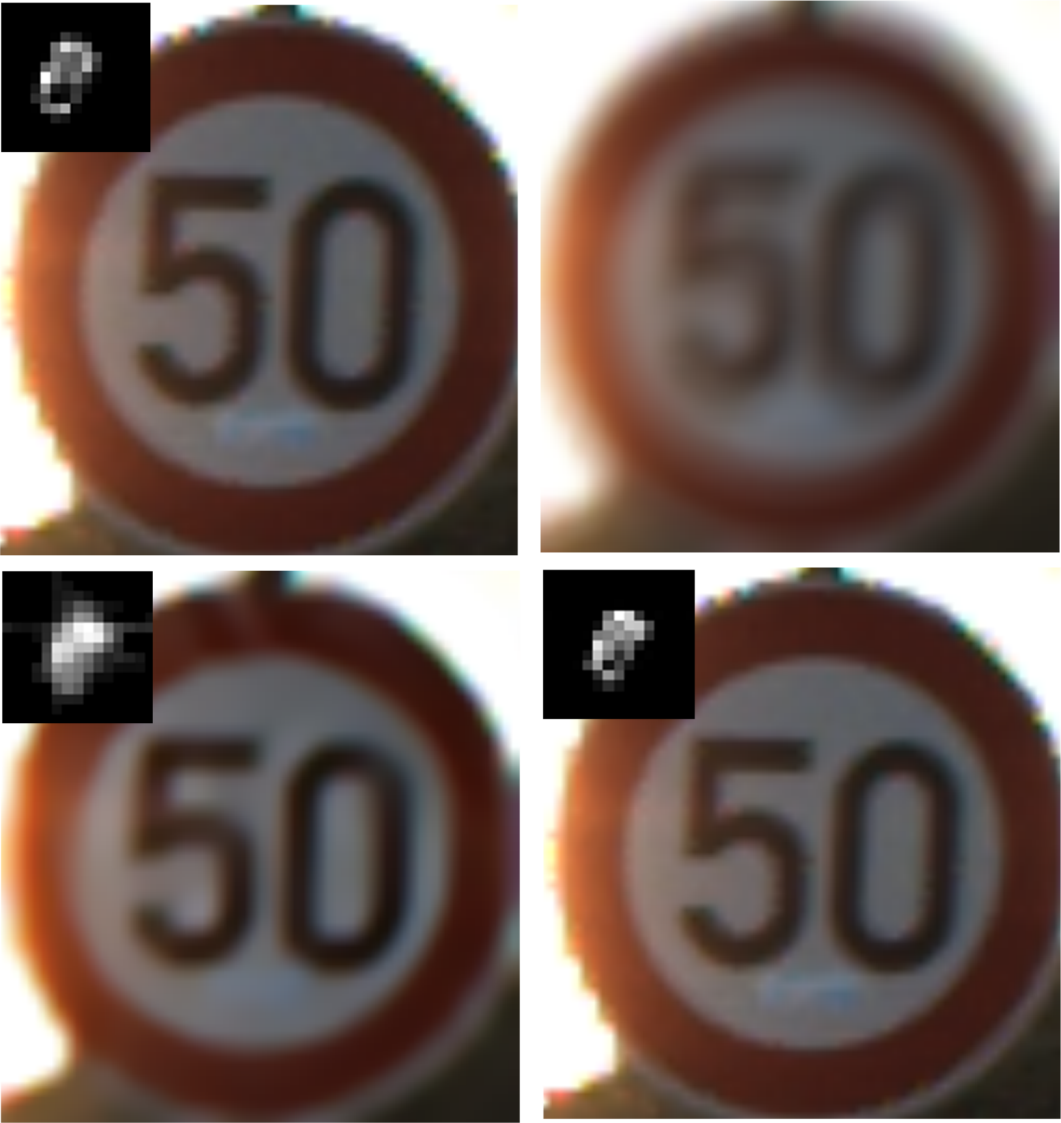
Deblurring results for synthetic traffic signs during late morning. (a) denotes the input image and real blur kernel. (b) denotes the blurred image. (c) and (d) denote the deblurring results of Pan[9] and our method, respectively.

**Fig 14:**
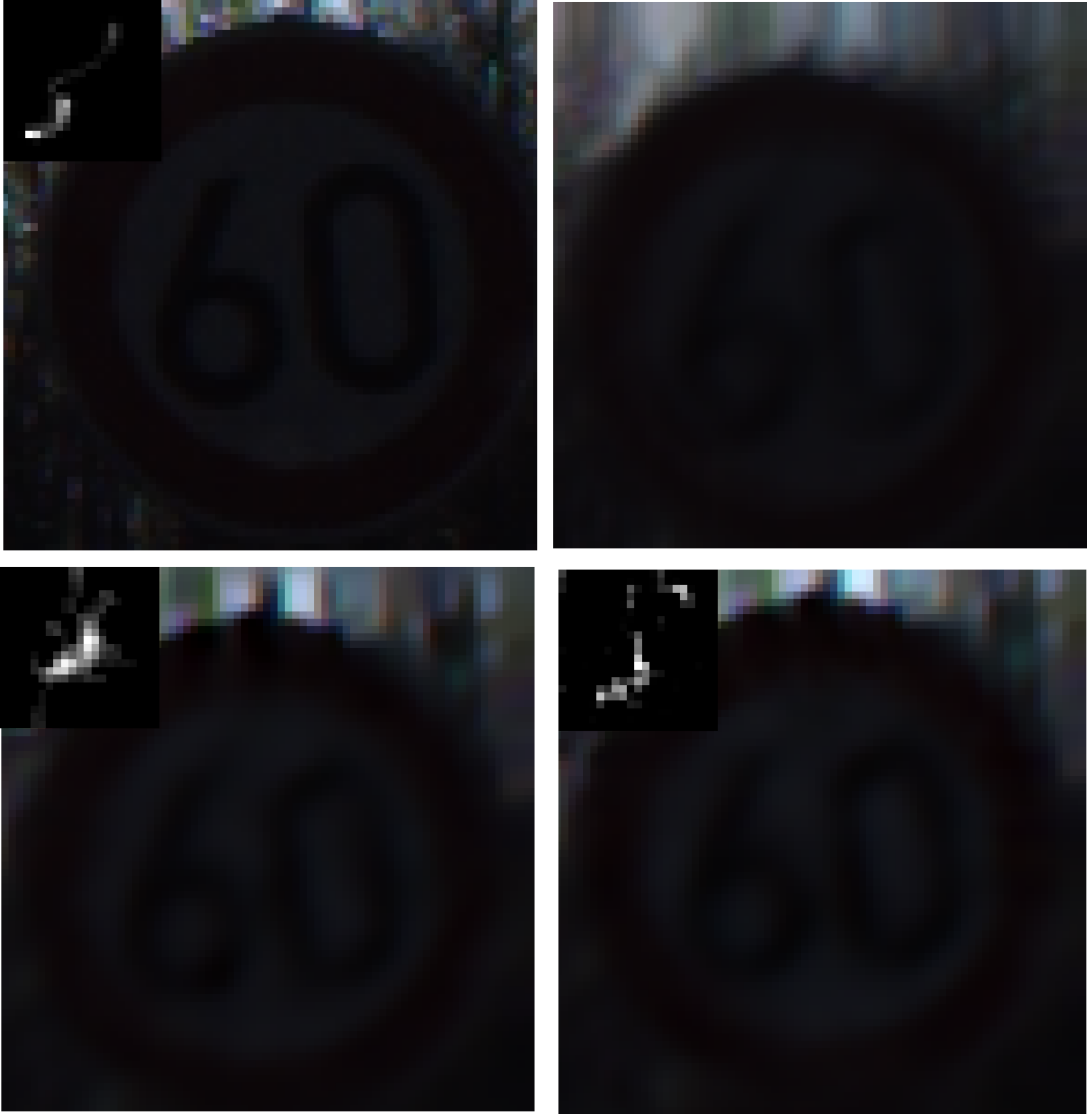
Deblurring results for synthetic traffic signs during early evening. (a) denotes the input image and real blur kernel. (b) denotes the blurred image. (c) and (d) denote the deblurring results of Pan[9] and our method, respectively.

In this part, synthetic traffic sign images are employed to verify the performance of the proposed method.Figs 10–14 illustrate the deblurring results for synthetic traffic signs with different scenarios.

From Fig 10 to Fig 14, it is clearly illustrated that the estimated motion blur kernel is clearer than those estimated by [9] and the proposed method performs better under common scenarios.

## Conclusion and future work

A novel methodfor deblurring traffic sign image is proposed based on exemplars in this paper. Using multiple-size partition strategy, we propose a novel dataset construction method and it lowers the calculation time costsignificantly during exemplar matching. We also propose an exemplar matching criterion named GECC which combines both spatial information and ECC, and it can help to achieve better accuracy. During kernel estimation procedure, we introduce the regulation term with *L*_0.5_ norm to better the sparsity of the kernel.

Experiments verify the superiority of the proposed approaches and extensive evaluations with state-of-the-art deblurring algorithms demonstrate that the proposed algorithm is effective fordeblurring traffic sign images. The future work will focus on the judge criterion for image blurred degree and further improving real time.

## Supporting information

S1 Fig Some examples of motion blur in traffic sign images with different sizes. (TIF)

S2 Fig Exemplar images and the corresponding contour masks. (TIF)

S3 Fig Matching results comparison. (TIFF)

S4 Fig Kernel similarity comparison. (TIFF)

S5 Fig Sparsity comparison. (TIFF)

S6 Fig Quantitative comparisons among blind deblurring methods. (TIFF)

S7 Fig Image deblurring comparison for synthetic images. (TIFF)

S8 Fig Deblurring example suffering from a heavier blur. (TIFF)

S9 Fig Example of real captured image. (TIFF)

S10 Fig Deblurring results for synthetic traffic signs during sunny day time. (TIF)

S11 Fig Deblurring results for synthetic traffic signs during cloudy day time. (TIF)

S12 Fig Deblurring results for synthetic traffic signs during foggy day time. (TIF)

S13 Fig Deblurring results for synthetic traffic signs during late morning. (TIF)

S14 Fig Deblurring results for synthetic traffic signs during early evening. (TIF)

S1 Table Matching calculation cost comparison. (PDF)

S2 Table Matching accuracy comparison. (PDF)

S1 Dataset The German Traffic Sign Recognition Benchmark. Available from: http://benchmark.ini.rub.de/?section=gtsrb&subsection=dataset.
(PDF)

## References

[1] Fleyeh H, Dougherty M. Road and traffic sign detection and recognition. Journal of Veterinary Medicine. 2005; 52(6):278–283. doi: 10.1111/j.1439-0450.2005.00866.x16219091

[2] Chourasia K, Chourasia J N. A Review and Comparative Analysis of Recent Advancements in Traffic Sign Detection and Recognition Techniques. SAMRIDDHI: A JOURNAL OF PHYSICAL SCIENCES, ENGINEERING AND TECHNOLOGY. 2011; 2(1):27–34.

[3] Fu M Y, Huang Y S. A survey of traffic sign recognition. 2010 International Conference on Wavelet Analysis and Pattern Recognition (ICWAPR). IEEE. 2010; 119–124.

[4] Gomez-Moreno H, Maldonado-Bascon S, Gil-Jimenez P, Lafuente-Arroyo S. Goal evaluation of segmentation algorithms for traffic sign recognition. IEEE Transactions on Intelligent Transport System. 2010; 11(4): 917–930.

[5] Lee S, Cho S. Recent Advances in Image Deblurring. SIGGRAPH Asia 2013 Course; 2013.

[6] Jia J. Mathematical models and practical solvers for uniform motion deblurring]. Cambridge University Press; 2014.

[7] Xu L, Jia J. Two-phase kernel estimation for robust motion deblurring. European Conference on Computer Vision. 2010; 157–170.

[8] Pan JS, Liu RS, Su ZX, Gu XF. Kernel Estimation from Salient Structure for Robust Motion Deblurring.Signal Processing: Image Communication. 2013; 28(9):1156–1170.

[9] Pan JS, Hu Z, Su ZX, Yang MH. Deblurring Face Images with Exemplars, European Conference on Computer Vision (ECCV). 2014; 47–62.

[10] Pan JS, Hu Z, Su ZX, Yang MH. L0-Regularized Intensity and Gradient Prior for Deblurring Text Images and Beyond. IEEE Transactions on Pattern Analysis and Machine Intelligence (TPAMI). 2017; 39(2): 342–355. 10.1109/TPAMI.2016.2551244 27071160

[11] Pan JS, Sun DQ, Pfister H, Yang MH. Blind Image Deblurring Using Dark Channel Prior. IEEE Conference on Computer Vision and Pattern Recognition(CVPR). 2016; 1628–1636.

[12] Krishnan D, Tay T, Fergus R. Blind Deconvolution using a Normalized Sparsity Measure. IEEE Conference on Computer Vision and Pattern Recognition. 2011; 2657–2664.

[13] Michaeli T, Irani M. Blind Deblurring Using Internal Patch Recurrence. European Conference on Computer Vision. 2014; 783–798.

[14] Levin A, Weiss Y, Durand F, Freeman WT. Understanding and evaluating blind deconvolution algorithms. IEEE Conference on Computer Vision and Pattern Recognition. 2009; 1964–1971.

[15] Shan Q, Jia J, Agarwala A. High-quality motion deblurring from a single image. ACMTransactionson Graphics. 2008; 27(3):73.

[16] Levin A, Weiss Y, Durand F, Freeman WT. Efficient Marginal Likelihood Optimization in Blind Deconvolution. IEEE Conference on Computer Vision and Pattern Recognition. 2011; 2657–2664.

[17] Xu L., Zheng S., Jia J. Unnatural L0 sparse representation for natural image deblurring. In: CVPR 2013; 1107–1114.

[18] Jia J. Single image motion deblurring using transparency. IEEE Conference on Computer Visionand Pattern Recognition. 2007; 1–8.

[19] Joshi N, Szeliski R, Kriegman D J. PSF estimation using sharp edge prediction. IEEE Conference on Computer Vision and Pattern Recognition. 2008; 1–8.

[20] Cho S, Lee S. Fast motion deblurring. ACM Transactions on Graphics. 2009; 28(5):145.

[21] HaCohen Y, Shechtman E, Lischinski D. Deblurring by Example using Dense Correspondence. International Conference on Computer Vision. 2013; 2384–2391.

[22] The German Traffic Sign Detection Benchmark, available from: http://benchmark.ini.rub.de/?section=gtsdb&subsection=dataset.

[23] He K, Sun J, Tang X. Guided Image Filtering. European Conference on Computer Vision. 2010; 1–14

[24] Otsu N. A Threshold Selection Method from Gray-Level Histograms. IEEE Transactions on Systems, Man, and Cybernetics. 1979; 9(9):62–66.

[25] Maes F, Collignon A, Vandermeulen D, etal Multimodality image registration by ma-ximization of mutual information. IEEE Transactions on Medical Imaging. 1997; 16(2):187–198. doi: 10.1109/42.563664 9101328 10.1109/42.563664

[26] Pluim J P W, Maintz J B A, Viergever M A. Image Registration by Maximization of Combined Mutual Information and Gradient Information. IEEE Transactions on Medical Imaging. 2000; 19(8): 809–814. doi: 10.1109/42.876307 11055805 10.1109/42.876307

[27] Xu L, Lu C, Xu Y, Jia J. Image smoothing via L_0_ gradient minimization. ACM Transactions on Graphics. 2011;30:174.

[28] Levin A., Fergus R., Durand F., Freeman B. Image and depth from a conventional camera with a coded aperture. ACM Trans. Graph,2007;26(3):70.

[29] Stallkamp J, Schlipsing M, Salmen J, lgel C. Man vs. computer: benchmarking machine learning algorithms for traffic sign recognition. Neural Networks. 2012; 32(2):323–332. 22394690 10.1016/j.neunet.2012.02.016

