# Supplementary figures and images for: Deblurring traffic sign images based on exemplars
Source: PLoS One. 2018 Mar 7;13(3):e0191367. doi: 10.1371/journal.pone.0191367 (PMC5841653; doi:10.1371/journal.pone.0191367)

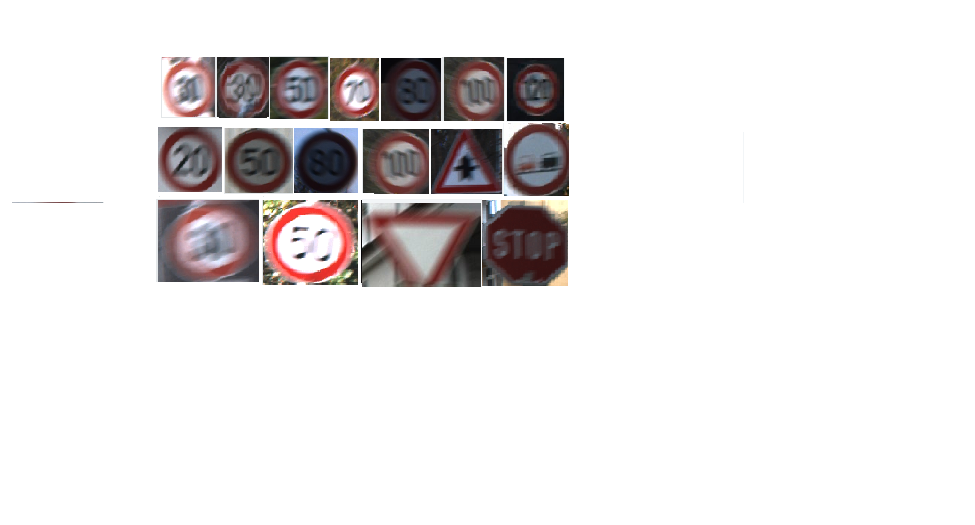

Supplement: S1 Fig — (TIF) [file pone.0191367.s001.tif]

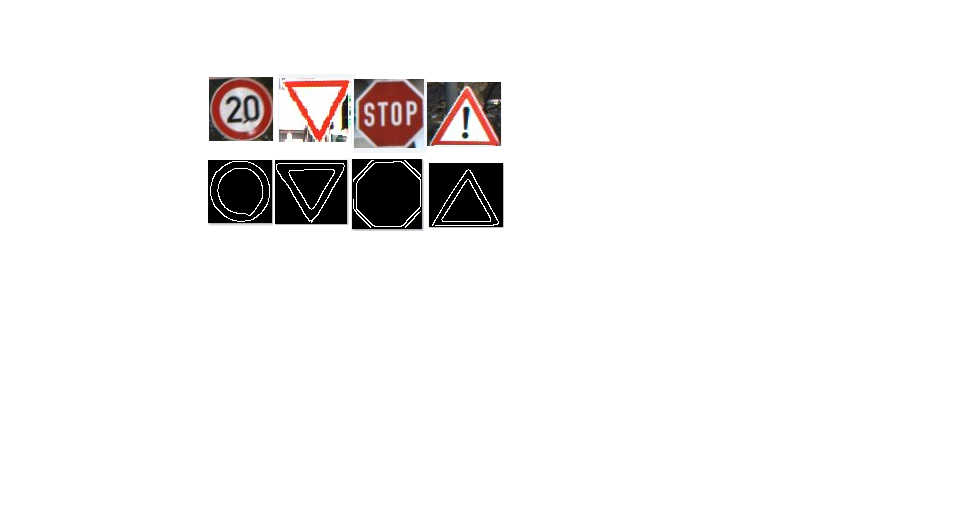

Supplement: S2 Fig — (TIF) [file pone.0191367.s002.tif]

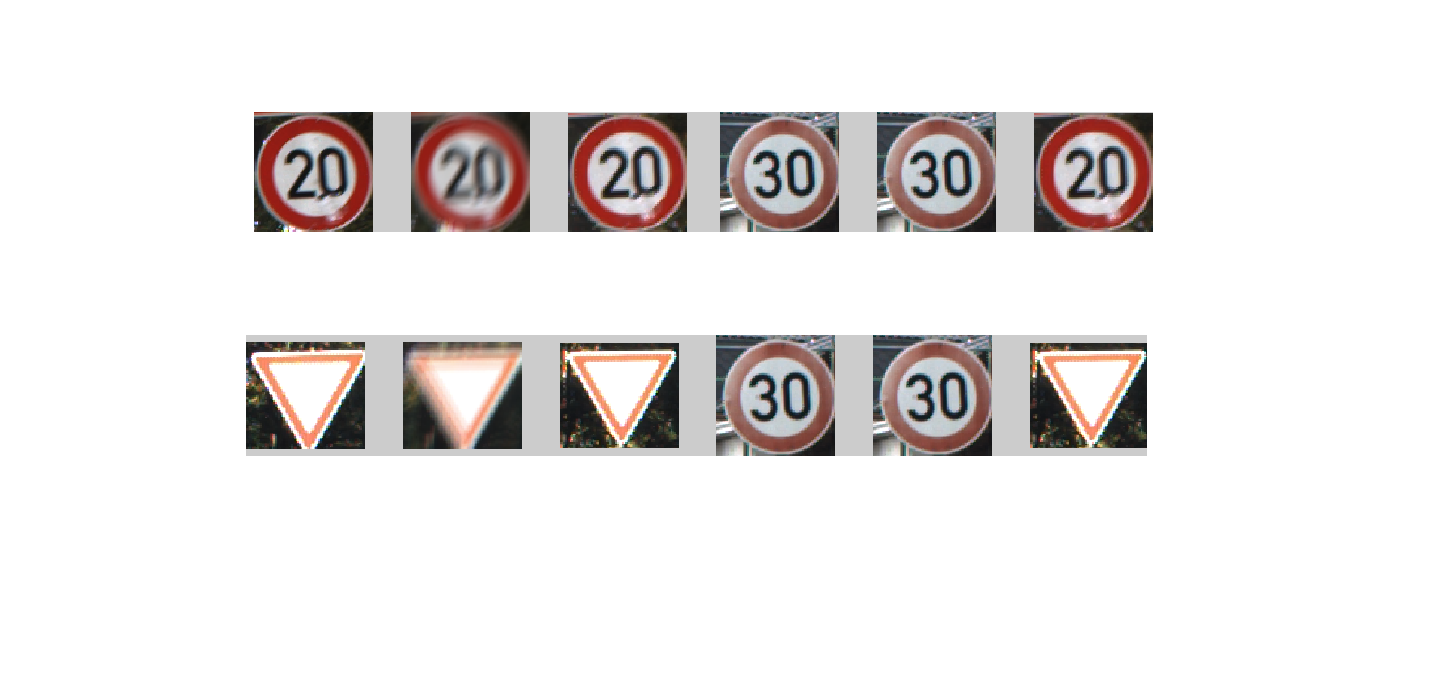

Supplement: S3 Fig — (TIFF) [file pone.0191367.s003.tiff]

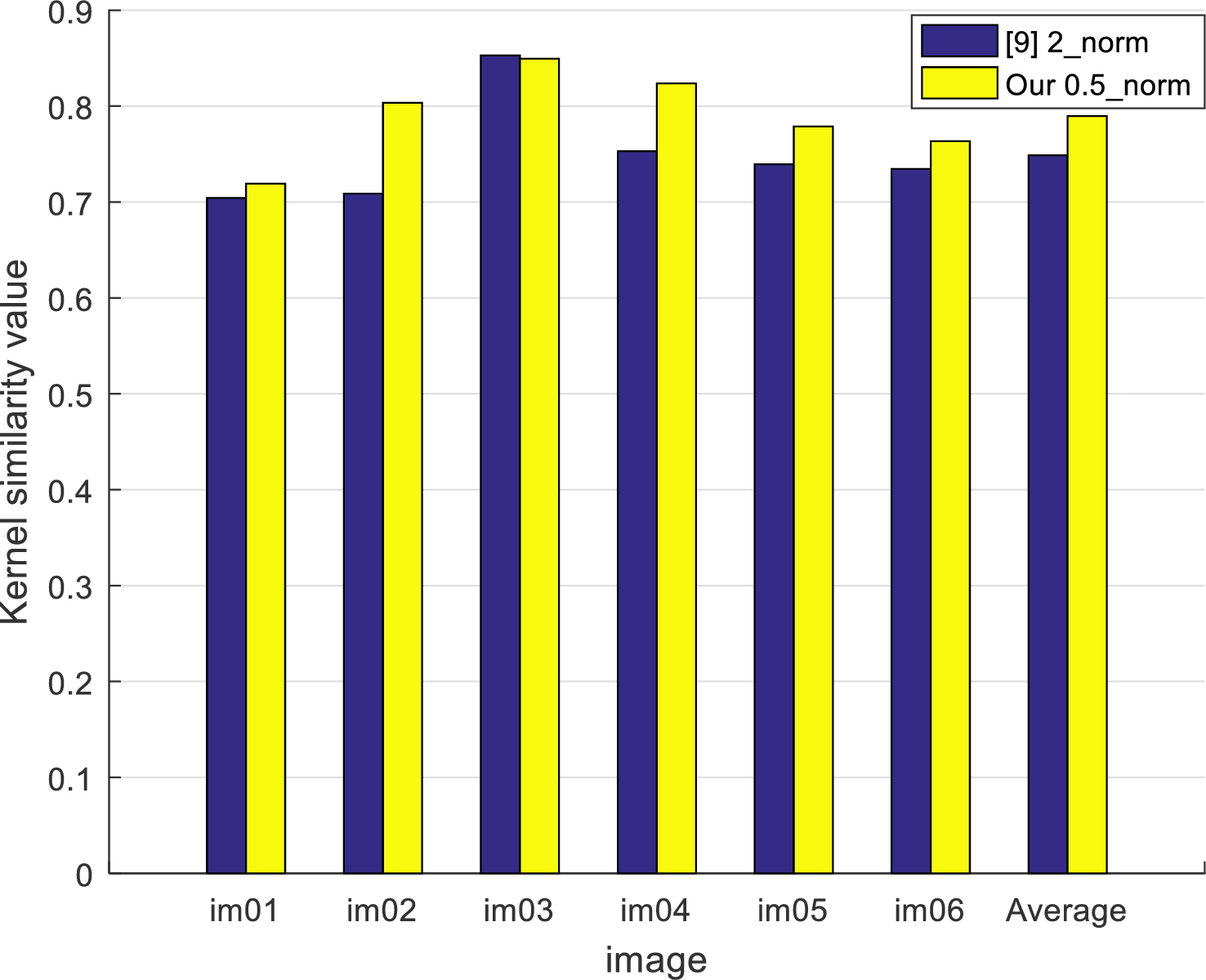

Supplement: S4 Fig — (TIFF) [file pone.0191367.s004.tiff]

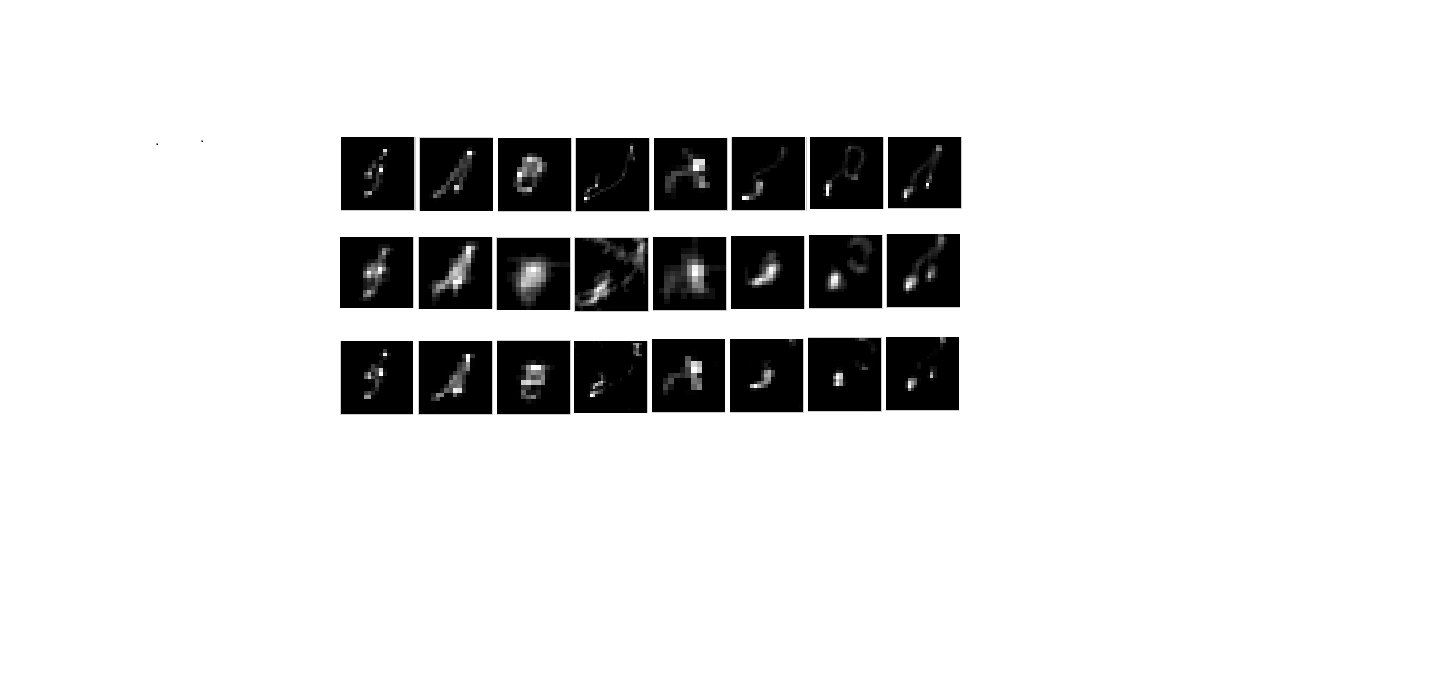

Supplement: S5 Fig — (TIFF) [file pone.0191367.s005.tiff]

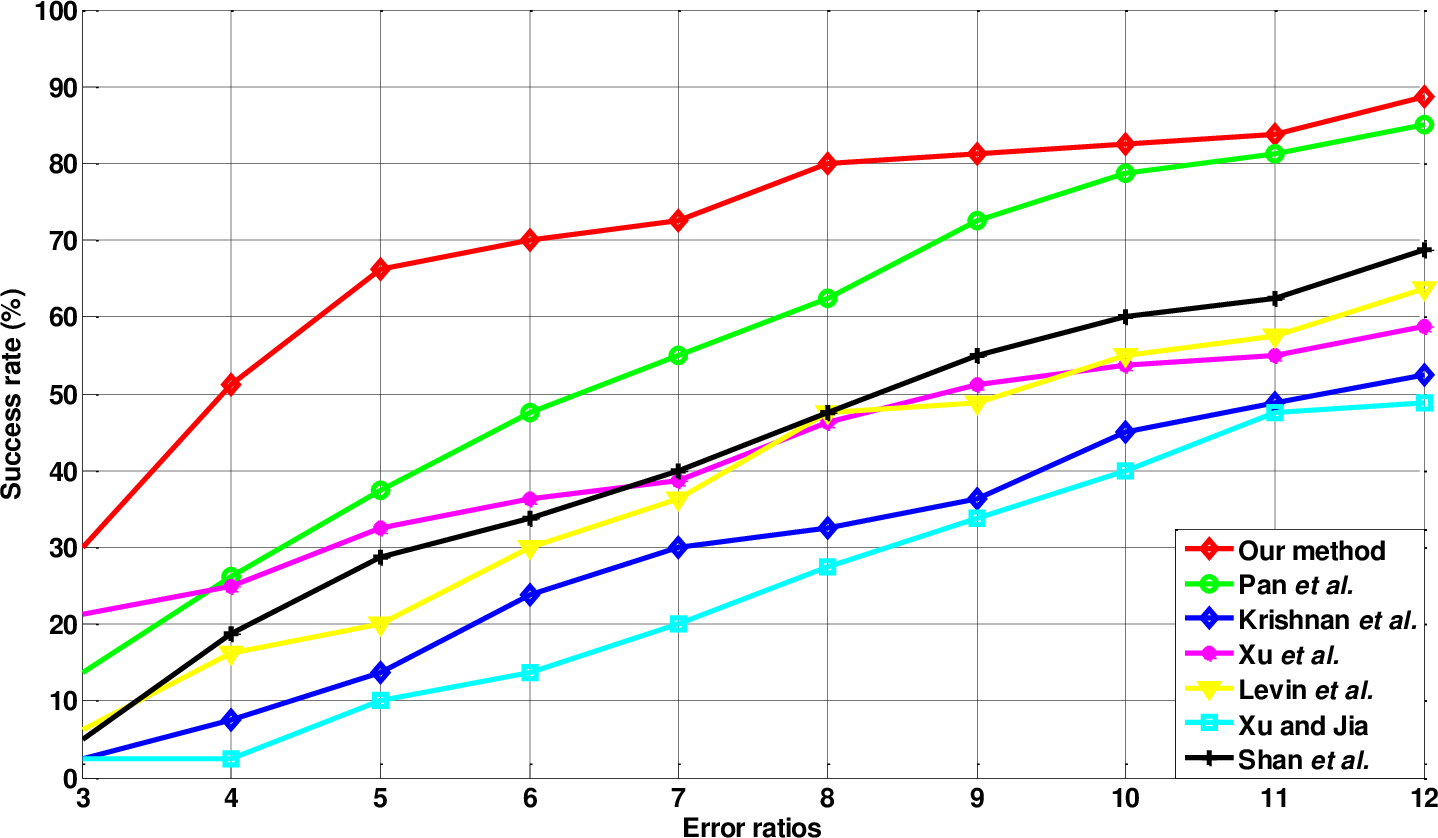

Supplement: S6 Fig — (TIFF) [file pone.0191367.s006.tiff]

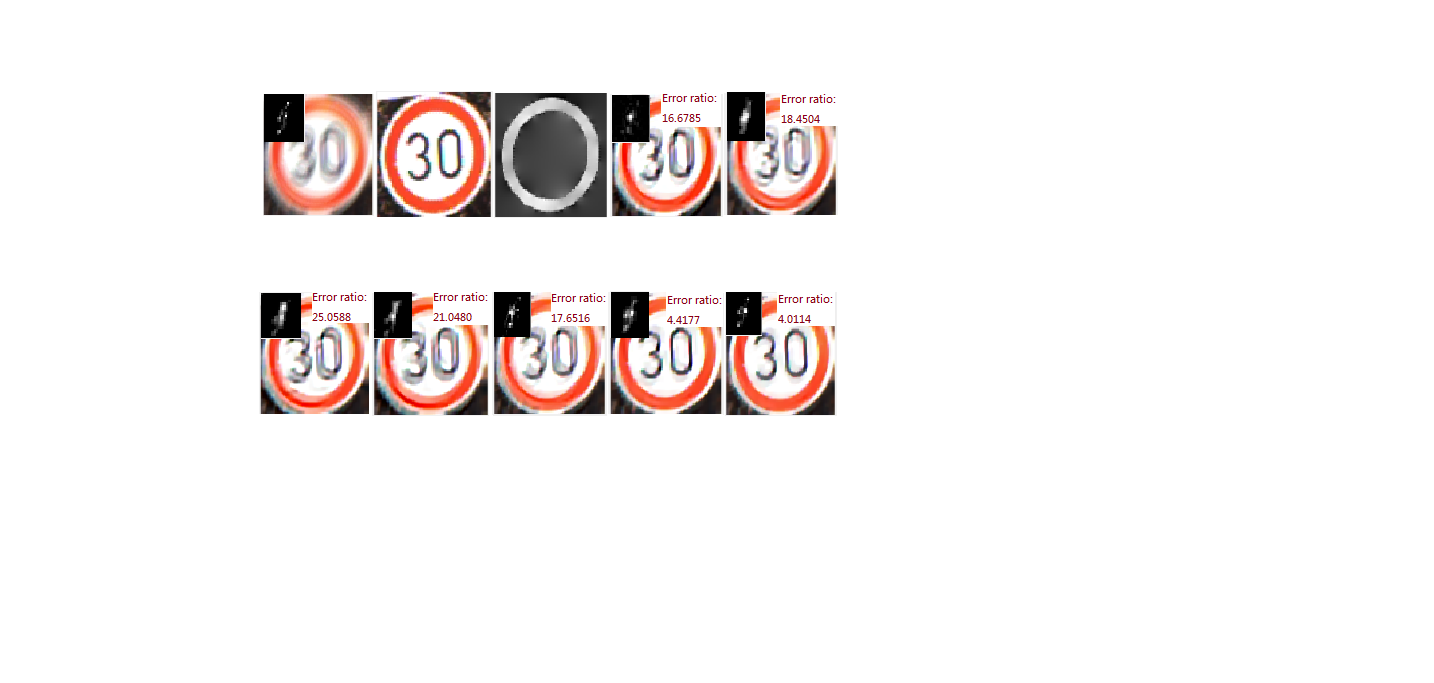

Supplement: S7 Fig — (TIFF) [file pone.0191367.s007.tiff]

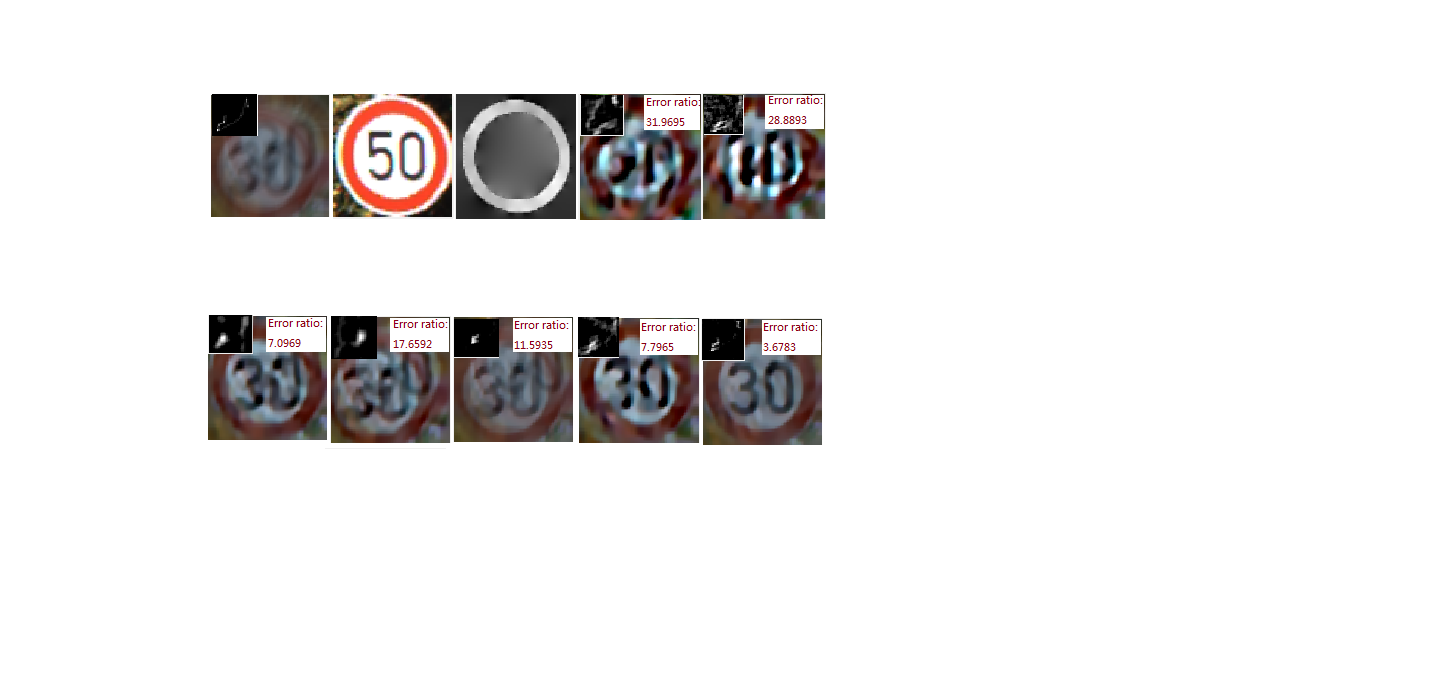

Supplement: S8 Fig — (TIFF) [file pone.0191367.s008.tiff]

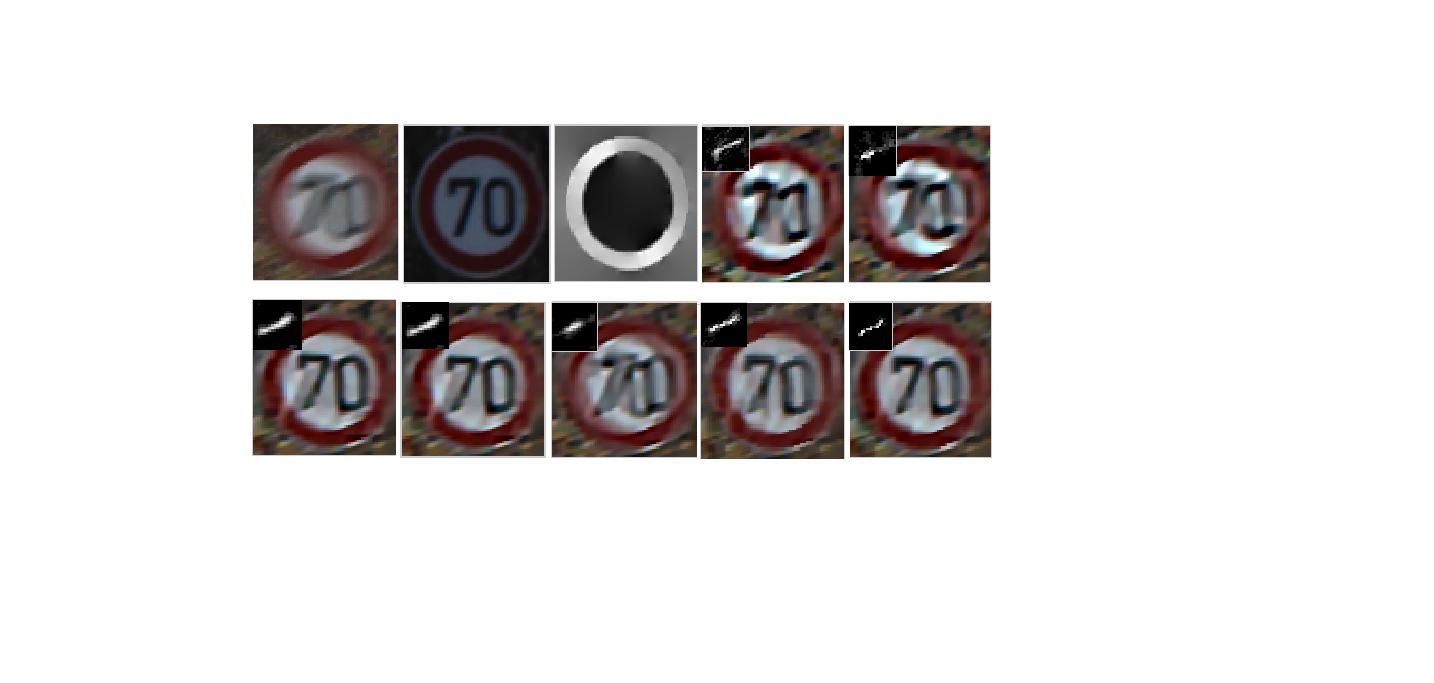

Supplement: S9 Fig — (TIFF) [file pone.0191367.s009.tiff]

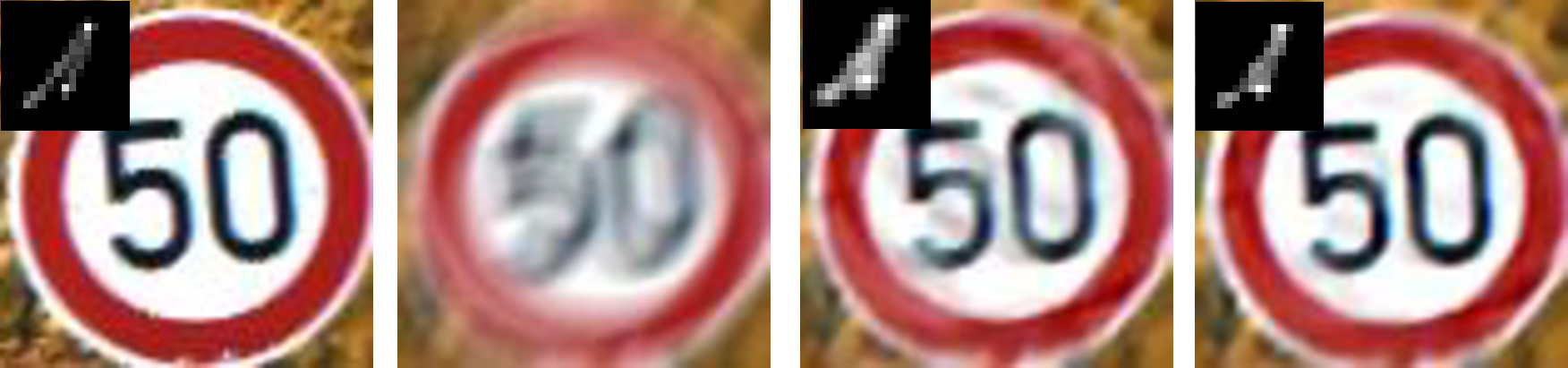

Supplement: S10 Fig — (TIF) [file pone.0191367.s010.tif]

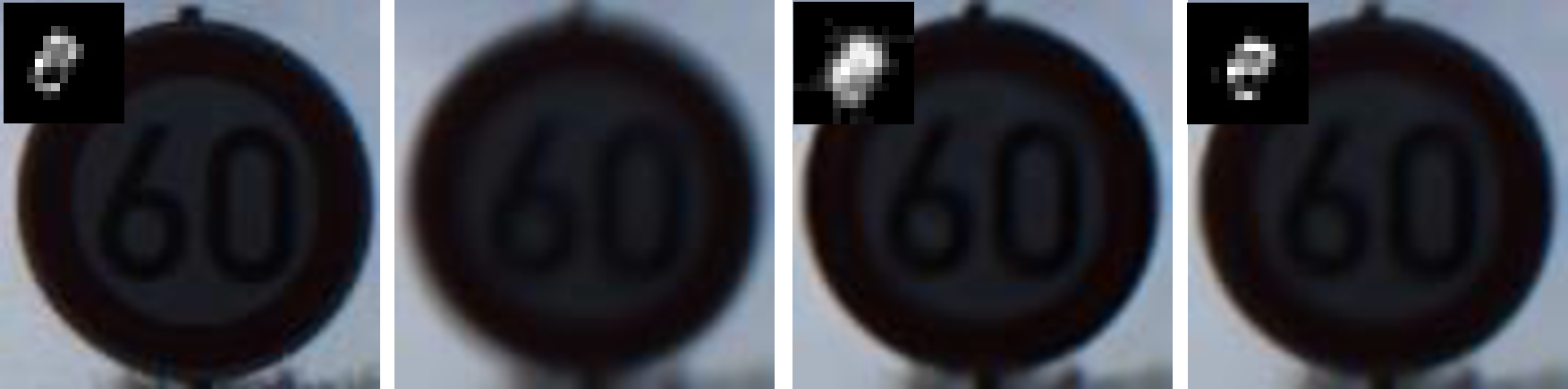

Supplement: S11 Fig — (TIF) [file pone.0191367.s011.tif]

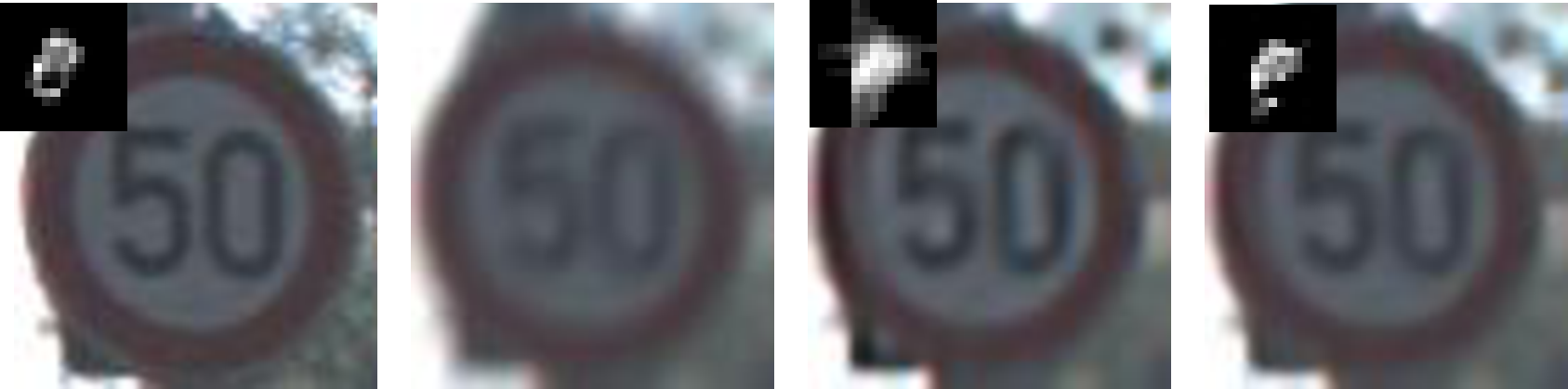

Supplement: S12 Fig — (TIF) [file pone.0191367.s012.tif]

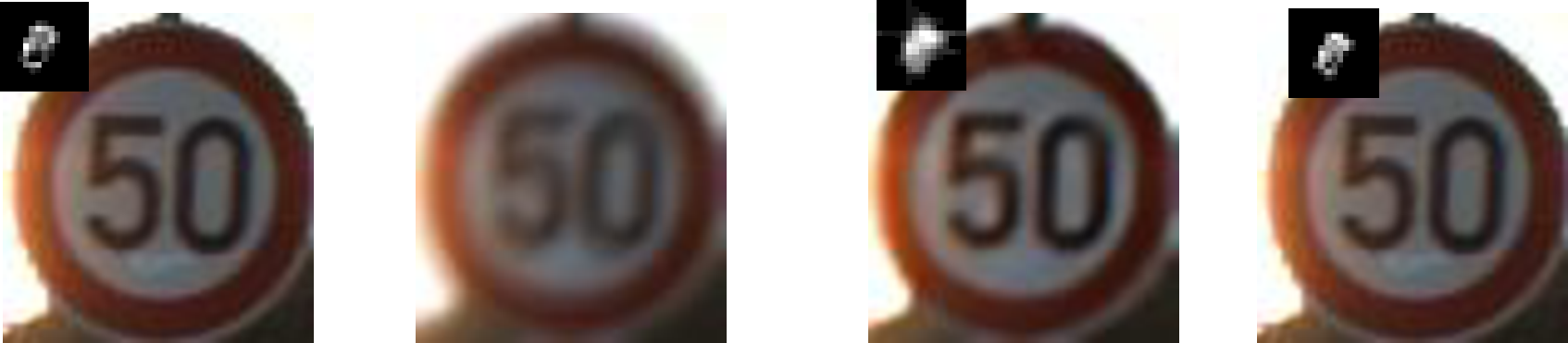

Supplement: S13 Fig — (TIF) [file pone.0191367.s013.tif]

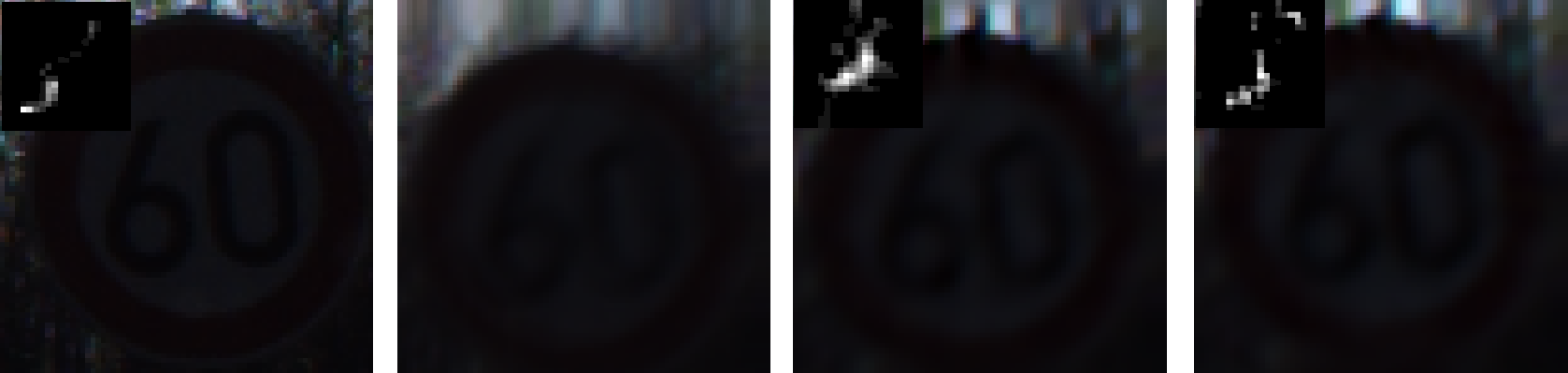

Supplement: S14 Fig — (TIF) [file pone.0191367.s014.tif]
