# Supplementary material for: Deblurring traffic sign images based on exemplars
Source: PLoS One. 2018 Mar 7;13(3):e0191367. doi: 10.1371/journal.pone.0191367 (PMC5841653; doi:10.1371/journal.pone.0191367)
Supplement: S1 Table — (PDF) [file pone.0191367.s015.pdf]

Table 1. Matching calculation cost comparison

methods	Test images	Exemplars involved in matching	Tatol calculation time(s)	Everage time of each image(s)
**[9]**	80	650	9380.7667	117.2599
**This paper**	80	50	662.0180	8.2752
