# Supplementary material for: Deblurring traffic sign images based on exemplars
Source: PLoS One. 2018 Mar 7;13(3):e0191367. doi: 10.1371/journal.pone.0191367 (PMC5841653; doi:10.1371/journal.pone.0191367)
Supplement: S2 Table — (PDF) [file pone.0191367.s016.pdf]

Table 2. Matching accuracy comparison

**Matching methods**	N_t	N_m	r_s
**NCC**	4400	4303	97.80%
**MI**	4400	3526	80.13%
**ECC**	4400	3789	86.11%
**GECC**	4400	4354	98.95%
