# Supplementary material for: Deblurring traffic sign images based on exemplars
Source: PLoS One. 2018 Mar 7;13(3):e0191367. doi: 10.1371/journal.pone.0191367 (PMC5841653; doi:10.1371/journal.pone.0191367)
Supplement: S1 Dataset — Available from: http://benchmark.ini.rub.de/?section=gtsrb&subsection=dataset. (PDF) [file pone.0191367.s017.pdf]

S1 Dataset. The German Traffic Sign Recognition Benchmark. Available from**:**

http://benchmark.ini.rub.de/?section=gtsrb&subsection=dataset
